# Two New Diketomorpholine Derivatives and a New Highly Conjugated Ergostane-Type Steroid from the Marine Algal-Derived Endophytic Fungus *Aspergillus* *alabamensis* EN-547

**DOI:** 10.3390/md16040114

**Published:** 2018-03-31

**Authors:** Sui-Qun Yang, Xiao-Ming Li, Xin Li, Lu-Ping Chi, Bin-Gui Wang

**Affiliations:** 1 Key Laboratory of Experimental Marine Biology, Institute of Oceanology, Chinese Academy of Sciences and Laboratory of Marine Biology and Biotechnology, Qingdao National Laboratory for Marine Science and Technology, Nanhai Road 7, Qingdao 266071, China; suiqunyang@163.com (S.-Q.Y.); lixmqdio@126.com (X.-M.L.); lixin871014@163.com (X.L.); 1061825010@qq.com (L.-P.C.); 2 University of Chinese Academy of Sciences, Yuquan Road 19A, Beijing 100049, China

**Keywords:** algal-derived endophytic fungus, *Aspergillus alabamensis*, diketomorpholine, steroid, antimicrobial activity

## Abstract

Chemical investigation of the marine algal-derived endophytic fungus *Aspergillus alabamensis* EN-547 resulted in the isolation of 4-*epi*-*seco*-shornephine A methyl ester (**1**) and 4-*epi*-*seco*-shornephine A carboxylic acid (**2**), two new secondary metabolites having a rare diketomorpholine motif, and 28-acetoxy-12*β*,15*α*,25-trihydroxyergosta-4,6,8(14),22-tetraen-3-one (**3**), a new highly conjugated ergostane-type steroid, together with four known metabolites (**4**–**7**). Their chemical structures were elucidated by detailed analysis of their NMR spectra, ECDs, HRESIMS, optical rotation, and X-ray crystallographic data, and by comparison with literature data as well. The antimicrobial activities of compounds **1**–**7** were evaluated.

## 1. Introduction

**Figure 1:**
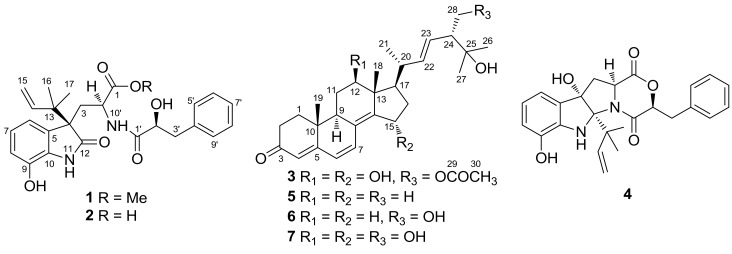
Structures of compounds **1**–**7**.

Marine-derived filamentous fungi, especially those of the species in the genera *Aspergillus* and *Penicillium*, are rich sources of natural products possessing diverse scaffolds and bioactivities [1]. Among them, fungi associated with marine algae are a promising source of research on the secondary metabolites. During our ongoing investigations of marine algal-derived fungi for structurally unique and biologically active metabolites [2,3,4], the fungal strain *Aspergillus alabamensis* EN-547 was isolated from the fresh inner tissue of the marine red alga *Ceramium japonicum*. When the EtOAc extracts of its culture broth was analyzed by HPLC (Figure S1 in the Supplementary Materials), and a series of peaks with similar UV absorptions at 225, 247, and 297 nm were detected and no hits were found in our HPLC-UV database. We thus performed a large-scale fermentation of this fungus in liquid medium, leading to the isolation of two new diketomorpholine derivatives (**1** and **2**), and a new ergostane-type steroid, 28-acetoxy-12*β*,15*α*,25-trihydroxyergosta-4,6,8(14),22-tetraen-3-one (**3**), together with four known metabolites, shornephine A (**4**) [5,6,7], 25-hydroxyergosta-4,6,8(14),22-tetraen-3-one (**5**) [8], 25,28-dihydroxyergosta-4,6,8(14),22-tetraen-3-one (**6**) [9], and 12*β*,15*α*,25,28-tetrahydroxyergosta-4,6,8(14),22-tetraen-3-one (**7**) [10] (Figure 1). The structures of these compounds were elucidated by detailed analysis of their spectroscopic data and the absolute configuration of compounds **1** and **3** were confirmed by X-ray diffraction analysis and the absolute structure of compound **2** was determined by comparison of ECD and optical rotation with that of compound **1**. Herein, we report the isolation, structure assignment, and biological evaluation of the isolated compounds.

## 2. Results and Discussion

### 2.1. Structure Elucidation of the New Compounds

**Figure 2:**
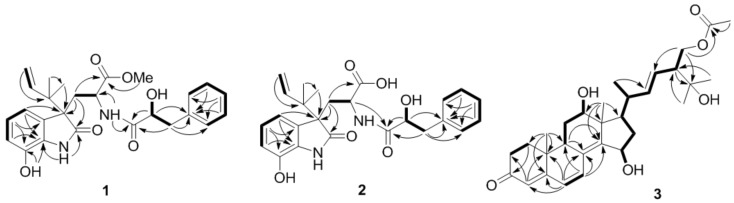
Key COSY (bold lines) and HMBC (arrows) correlations for compounds **1**–**3**.

**Figure 3:**
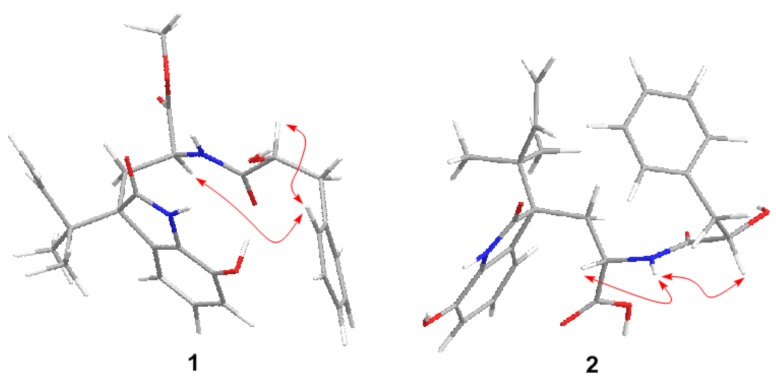
Key NOESY correlations for compounds **1** and **2**.

**Figure 4:**
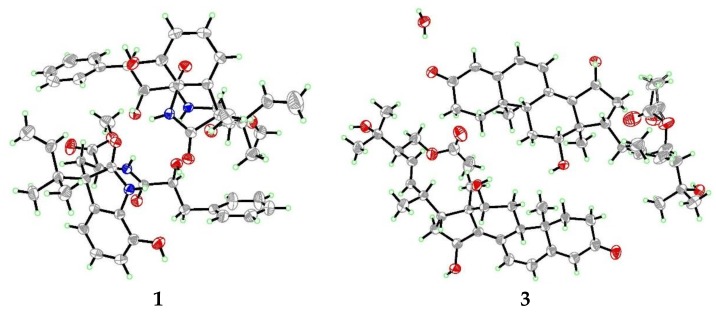
X-ray crystallographic structures of compounds **1** and **3**.

Compound **1** was initially isolated as a white amorphous powder. The positive ion ESIMS spectrum showed four peaks at *m*/*z* 467.22 [M + H]^+^, 489.20 [M + Na]^+^, 933.43 [2M + H]^+^, and 955.41 [2M + Na]^+^, and its molecular formula was assigned as C_26_H_30_N_2_O_6_ by HRESIMS at *m*/*z* 467.2168 [M + H]^+^ (calcd. 467.2177), indicating 13 degrees of unsaturation. The ^1^H NMR data of **1** (Table 1) indicated the resonances corresponding to three aliphatic methyls (with one methoxy), three methylenes (with two aliphatic and one olefinic), 11 methines (with one olefinic, two aliphatic, and eight aromatic), and four exchangeable protons. The ^13^C NMR and DEPT spectra revealed the presence of 26 carbons that were assigned as three methyls, three methylenes, 11 methines, and nine quaternary carbons. All of the ^1^H and ^13^C NMR data were quite similar to those of previously reported diketomorpholine derivative, *seco*-shornephine A methyl ester [6]. Interpretation of the COSY and HMBC correlations (Figure 2) revealed that the planar of **1** is same as that of *seco*-shornephine A methyl ester. However, NOESY experiments only yielded two NOEs from H-2 and H-2′ to H-5′/9′ (Figure 3), which could not fully assign the relative configuration of **1**. Upon slow evaporation of **1** in a mixture of methanol and EtOAc, quality crystals suitable for X-ray experiments were obtained. Discreet analysis of the crystallographic data of compound **1** (Figure 4) revealed that the absolute configuration of **1** at C-4 was different from that of *seco*-shornephine A methyl ester. The Cu K*α* Flack parameter 0.0(2) of **1** allowed unambiguously confirmation of the absolute configurations of **1** as 2*S*, 4*R*, and 2′*S*, and the trival name 4-*epi-seco*-shornephine A methyl ester was assigned to compound **1**.

**Figure 5:**
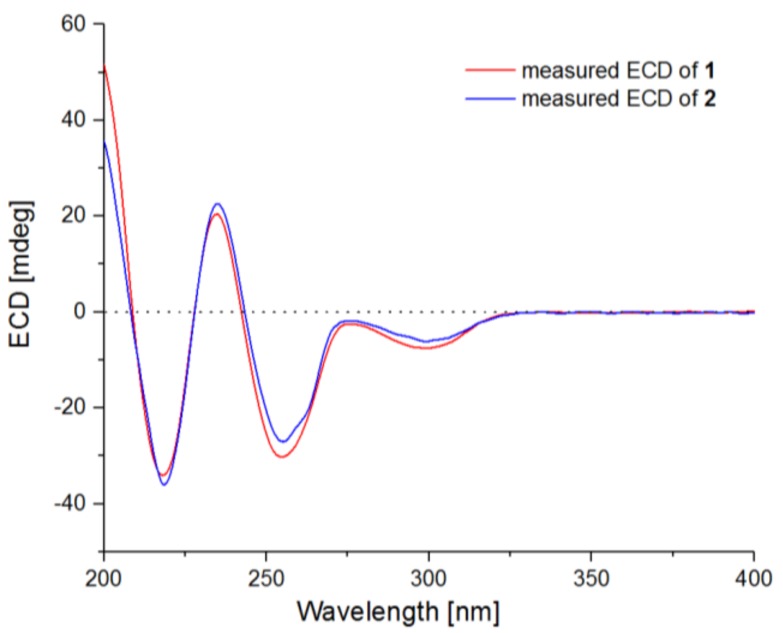
ECD spectra of compounds **1** and **2**.

Compound **2** was obtained as a pale yellow gum and had the molecular formula C_25_H_28_N_2_O_6_, having one CH_2_ unit less than that of **1**, as determined by HRESIMS at *m*/*z* 453.2018 [M + H]^+^ (calcd. 453.2020). The ^1^H and ^13^C NMR data (Table 1) of **2** were very similar to those of **1**. However, signals for the methyl group in the methyl ester moiety of **1** disappeared in that of **2**, and the chemical shift of C-1 deshielded from δ_C_ 172.1 in **1** to 180.2 in **2**, indicating that compound **1** was hydrolyzed to form a carboxylic acid in **2**. In the NOESY experiment, both H-2 and H-2′ showed correlations to H-10′ (Figure 3), which could also not be able to fix the relative configuration of **2**. However, from a biosynthetic point of view, compound **2** should possesses the same configuration as that of **1**, and this hypothesis was supported by the fact that the optical rotation of compound **2** ([α${]}_{D}^{25}$ −61.8 (*c* 0.25, MeOH)) has same sign as that of **1** ([α${]}_{D}^{25}$ −115.8 (*c* 0.19, MeOH)). Furthermore, the ECD spectrum of compound **2** exhibited Cotton effects (CEs) virtually identical to those of **1**, which showed positive CE at 236 nm and negative CEs near 219, 256, and 300 nm (Figure 5). Based on the above discussion, the structure of compound **2** was determined and the trivial name 4-*epi-seco*-shornephine A carboxylic acid was assigned.

The analysis of both of the original EtOAc extracts of *A. alabamensis* EN-547 and the purified metabolites **1** and **2** were carried out by our standard HPLC program eluting with MeOH-H_2_O, and no acid such as HCl or CH_3_COOH or CF_3_COOH was added in the solvent system. Compounds **1** and **2** had similar retention times and UV absorptions (Figures S8 and S17) to those of the corresponding peaks in the HPLC profile of the original EtOAc extracts (Figure S1), suggesting that compounds **1** and **2** were the natural metabolites of this fungus. Aparicio-Cuevas and co-workers had similar conclusion to this kind of compounds [7].

**Figure 6:**
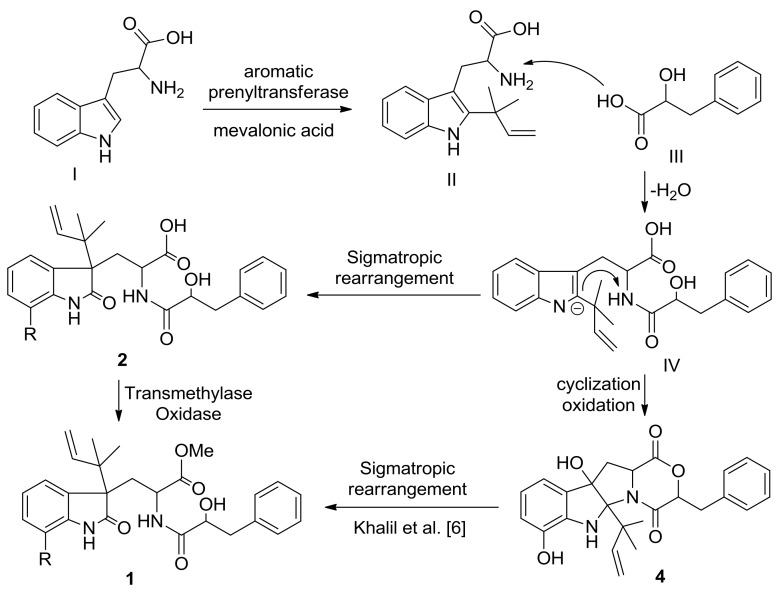
Proposed biosythesis of diketomorpholine derivatives **1** and **2**.

The biosynthesis of diketomorpholine derivatives **1** and **2** were proposed as shown in Figure 6. Tryptophan (I) is considered as the starting material that is prenylated to form an intermediate (II), followed by addition with β-hydroxyphenylalanic acid (III) to form the known metabolite, 4,9-dideoxy-seco-PF1233 B carboxylic acid (IV) [7]. Compound **4** can be synthesized by immediately cyclization of IV, while compound **2** could be produced by a sigmatropic rearrangement of IV, following by a transmethylase oxidation to form compound **1**. Compound **1** might also be obtained as a methanolysis product of compound **4** [6].

Compound **3** was initially isolated as a white amorphous powder and deduced to possess the molecular formula C_30_H_42_O_6_ by HRESIMS at *m*/*z* 499.3048 [M + H]^+^ (calcd. 499.3054), implying 10 degrees of unsaturation. The ^1^H and ^13^C NMR spectrascopic data of **3** revealed the presence of six methyls (with one methoxy), five methylenes (with one oxygenated), 11 methines (with two oxygenated and five olefinic), and eight quaternary carbons (Table 2). Detailed analysis of its 1D and 2D NMR data (Figure 2) suggested that **3** shared the same carbon skeleton as that of 12*β*,15*α*,25,28-tetrahydroxyergosta-4,6,8(14),22-tetraen-3-one (**7**) [10], except for the observation of signals for one additional ester carbonyl carbon (δ_C_ 170.3, C-29) and for a methyl group (δ_C_/δ_H_ 20.7/1.93, C-30/H-30), both of which belonged to an acetoxy group in **3**. The acetoxy group was located at C-28 as evidenced by HMBC correlation from H-28 to C-29 (Figure 2). The NOESY spectrum of **3** lacked critical data for assignment of its relative configurations. Nevertheless, a suitable rhombic crystal of **3** was acquired after low temperature evaporation of methanol and was applied to X-ray diffraction experiment using Cu K*α* radiation. As a result, both of the relative and absolute configurations of **3** were thus determined, and the refined Flack parameter 0.0(3) allowed the demonstrable stereochemical assignment of **3** as 9*R*, 10*R*, 12*R*, 13*R*, 15*S*, 17*R*, 20*R*, and 24*R* (Figure 4). On the basis of the above data, the chemical structure of compound **3** was assigned as 28-acetoxy-12*β*,15*α*,25-trihydroxyergosta-4,6,8(14),22-tetraen-3-one.

### 2.2. Biological Activities of the Isolated Compounds

Compounds **1**–**7** were tested for antimicrobial activities against two human pathogens (*Escherichia coli* and *Micrococcus luteus*) and five aquatic bacteria (*Edwardsiella ictaluri*, *Vibrio alginolyticus*, *V. anguillarum*, *V. parahaemolyticus*, and *V. vulnificus*). As shown in Table 3, compounds **1**–**4** showed inhibitions against human pathogens (*E. coli* and *M. luteus*) hand aquatic bacteria (*Ed. ictaluri* and *V. alginolyticus*), with MIC values ranging from 16 to 64 μg/mL. None of the tested compounds showed inhibitions against *V. anguillarum*, *V. parahaemolyticus*, and *V. vulnificus* (MIC > 64 μg/mL).

## 3. Experimental Section

### 3.1. General Experimental Procedures

Melting points were determined with an SGW X-4 micro-melting-point apparatus. Optical rotations were measured on an Optical Activity AA-55 polarimeter. UV spectra were measured on a PuXi TU-1810 UV-visible spectrophotometer. ECD spectra were acquired on a Chirascanspectropolarimeter. The ^1^H, ^13^C, and 2D NMR spectra were acquired using a Bruker Avance 500 spectrometer (Bruker Biospin Group, Karlsruhe, Germany). Mass spectra were determined on a VG Autospec3000 or an API QSTAR Pulsar 1 mass spectrometer. Analytical and semi-preparative HPLC were performed using a Dionex HPLC system equipped with P680 pump, ASI-100 automated sample injector, and UVD340U multiple wavelength detector controlled by Chromeleon software (version 6.80, Dionex, Sunnyvale, CA, USA). Column chromatography (CC) was performed with silica gel (200–300 mesh, Qingdao Haiyang Chemical Factory, Qingdao, China), Lobar LiChroprep RP-18 (40–60 μm, Merck, Darmstadt, Germany), and Sephadex LH-20 (18–110 μm, Merck).

### 3.2. Fungal Material

The fungal strain *A. alabamensis* EN-547 was isolated from the fresh inner tissue of the marine red alga *Ceramium japonicum* collected at Qingdao, China, in August 2016, using a protocol as described in our previous report [11]. Fungal identification was performed by analysis of its ITS region of the rDNA as described previously [11]. The resulting sequence data, which were most similar (99%) to the sequence of *A. alabamensis* (compared with KP987071), have been deposited in GenBank (accession no. MG461687). The strain is preserved at the Key Laboratory of Experimental Marine Biology, Institute of Oceanology of the Chinese Academy of Sciences (IOCAS).

### 3.3. Fermentation

For chemical investigations, the fresh mycelia of *A. alabamensis* EN-547 was grown on PDA medium at 28 °C for 6 days and then inoculated for 30 days at room temperature in 96 × 1 L conical flasks with MH2 medium (each flask contained 6.00 g mannitol, 6.00 g maltose, 3.00 g glucose, 3.00 g sodium glutamate, 0.90 g yeast extract, 0.30 g corn starch, 0.15 g K_2_HPO_4_, 0.09 g MgSO_4_·7H_2_O, and 300 mL naturally sourced and filtered seawater, which was obtained from the Huiquan Gulf of the Yellow Sea near the campus of IOCAS, pH 6.5−7.0).

### 3.4. Extraction and Isolation

The whole fermented cultures were filtered to separate the broth from the mycelia. The former was extracted with EtOAc for three times, while the latter was extracted three times with a mixture of 80% acetone and 20% H_2_O. The acetone solution was evaporated under reduced pressure to afford an aqueous solution, which was then extracted three times with EtOAc. Because the TLC and HPLC profiles of the two EtOAc extracts were almost identical, they were combined and concentrated under reduced pressure to afford an extract (46.30 g). The extract was fractionated by Si gel vacuum liquid chromatography (VLC) using different solvents of increasing polarity from petroleum ether (PE) to MeOH to yield nine fractions (Frs. 1–9) based on TLC and HPLC analysis. Purification of Fr. 4 (17.30 g) by reversed-phase column chromatography (CC) over Lobar LiChroprep RP-18 with a MeOH-H_2_O gradient (from 10:90 to 90:10) yielded nine subfractions (Frs. 4.1–4.9). Fr. 4.5 (1.6 g) was purified by CC on Sephadex LH-20 (MeOH) and then applied to preparative TLC (pTLC) (plate: 20 × 20 cm, developing solvents: CHCl_3_/MeOH, 30:1) to yield compound **1** (50.6 mg). Fr. 4.3 (700 mg) was purified by CC on Sephadex LH-20 (MeOH) and then applied to pTLC (developing solvents: PE/acetone, 5:1) to obtain compound **2** (96.3 mg). Fr. 4.6 (2.2 g) was set to CC on Si gel eluting with a PE–acetone gradient (from 30:1 to 1:1) and then purified by CC on Sephadex LH-20 (MeOH) to obtain compound **3** (7.2 mg) and compound **4** (28.4 mg). Fr. 4.7 (2.6 g) was purified by CC on Si gel eluting with a PE–ethyl acetate (EtOAc) gradient (from 30:1 to 1:1) and then applied to CC on Sephadex LH-20 (MeOH) to obtain compound **5** (20.5 mg), **6** (31.1 mg), and **7** (20.3 mg).

4-*epi*-*seco*-Shornephine A methyl ester (**1**): white, amorphous powder; mp 235–237 °C; [α${]}_{D}^{25}$ −115.8 (*c* 0.19, MeOH); UV (MeOH) *λ*_max_ (log *ε*) 219 (3.62), 248 (3.48), 299 (3.06); ECD (3.43 mM, MeOH) *λ*_max_ (Δ*ε*) 219 (−29.52), 236 (+17.47), 256 (−24.69), 300 (−5.41) nm; ^1^H and ^13^C NMR data, see Table 1; ESIMS at *m*/*z* 467.22 [M + H]^+^, 489.20 [M + Na]^+^, 933.43 [2M + H]^+^, and 955.41 [2M + Na]^+^; HRESIMS at *m*/*z* 467.2168 [M + H]^+^ (calcd. for C_26_H_31_N_2_O_6_, 467.2177).

4-*epi*-*seco*-Shornephine A carboxylic acid (**2**): pale yellow gum; [α${]}_{D}^{25}$ −61.8 (*c* 0.25, MeOH); UV (MeOH) *λ*_max_ (log *ε*) 220 (3.68), 247 (3.54), 298 (3.02) nm; ECD (7.08 mM, MeOH) *λ*_max_ (Δ*ε*) 219 (−15.38), 236 (+4.52), 256 (−21.87), 299 (−5.71) nm; ^1^H and ^13^C NMR data, see Table 1; ESIMS at *m*/*z* 453.20 [M + H]^+^; HRESIMS *m*/*z* 453.2018 [M + H]^+^ (calcd. for C_25_H_29_N_2_O_6_, 453.2020).

28-Acetoxy-12*β*,15*α*,25-trihydroxyergosta-4,6,8(14),22-tetraen-3-one (**3**): white, amorphous powder; mp 145–147 °C; [α${]}_{D}^{25}$ +470.0 (*c* 0.10, MeOH); UV (MeOH) *λ*_max_ (log *ε*) 256 (3.52), 336 (4.46) nm; ECD (1.81 mM, MeOH) *λ*_max_ (Δ*ε*) 360 (+66.37) nm; ^1^H and ^13^C NMR data, see Table 2; ESIMS at *m*/*z* 499.30 [M + H]^+^; HRESIMS *m*/*z* 499.3048 [M + H]^+^ (calcd. for C_30_H_43_O_6_, 499.3054).

### 3.5. X-ray Crystallographic Analysis of Compounds ***1*** and ***3*** *[12]*

All crystallographic data were collected on an Agilent Xcalibur Eos Gemini CCD plate diffractometer, using graphite monochromatized Cu/K*α* radiation (λ = 1.54178 Å). The data were corrected for absorption by using the program SADABS [13]. The structures were solved by direct methods with the SHELXTL software package (version 6.12, Göttingen, Germany) [14]. All non-hydrogen atoms were refined anisotropically. The H atoms were located by geometrical calculations, and their positions and thermal parameters were fixed during the structure refinement. The structure was refined by full-matrix least-squares techniques [15].

Crystal data for compound **1**: C_26_H_30_N_2_O_6_, F.W. = 466.52, Monoclinic space group P2(1), unit cell dimensions *a* = 11.9454(5) Å, *b* = 12.5452(4) Å, *c* = 32.4188(10) Å, *V* = 4858.2(3) Å^3^, *α* = *β* = *γ* = 90°, *Z* = 8, *d*_calcd_ = 1.276 mg/m^3^, crystal dimensions 0.37 × 0.35 × 0.18 mm, *μ* = 0.745 mm^–^^1^, *F*(000) = 1984. The 11,377 measurements yielded 7281 independent reflections after equivalent data were averaged, and Lorentz and polarization corrections were applied. The final refinement gave *R*_1_ = 0.0512 and w*R*_2_ = 0.1243 [*I* > 2*σ*(*I*)]. The Flack parameter was 0.0(2) in the final refinement for all 11,377 reflections with 7281 Friedel pairs.

Crystal data for compound **3**: C_30_H_42_O_6_, F.W. = 498.30, Monoclinic space group P2(1), unit cell dimensions *a* = 9.8131(6) Å, *b* = 18.7407(6) Å, *c* = 31.7360(13) Å, *V* = 5836.4(5) Å^3^, *α* = *β* = *γ* = 90°, *Z* = 8, *d*_calcd_ = 1.155 mg/m^3^, crystal dimensions 0.32 × 0.27 × 0.12 mm, *μ* = 0.644 mm^–^^1^, *F*(000) = 2200. The 13772 measurements yielded 8891 independent reflections after equivalent data were averaged, and Lorentz and polarization corrections were applied. The final refinement gave *R*_1_ = 0.0664 and w*R*_2_ = 0.1780 [*I* > 2*σ*(*I*)]. The Flack parameter was 0.0(3) in the final refinement for all 13,772 reflections with 8891 Friedel pairs.

### 3.6. Antimicrobial Assay

Antimicrobial evaluation against two human pathogens (*Escherichia coli* and *Micrococcus luteus*) and five aquatic bacteria (*Edwardsiella ictaluri*, *Vibrio alginolyticus*, *V. anguillarum*, *V. parahaemolyticus*, and *V. vulnificus*) was carried out by the microplate assay [16]. The human and aquatic pathogens were obtained from the Institute of Oceanology, Chinese Academy of Sciences. Chloramphenicol was used as a positive control.

## 4. Conclusions

In summary, we have identified three new compounds (**1**–**3**) and four related known natural products from the marine algal-derived endophytic fungus, *A. alabamensis* EN-547. Among them, compounds **1** and **2** are rare diketomorpholine derivatives, whereas compound **3** is a highly conjugated ergostane-type steroid. Compounds **1**–**4** showed inhibitions against human pathogens *E. coli* and *M. luteus* and aquatic bacteria *Ed. ictaluri* and *V. alginolyticus* with MIC values ranging from 16 to 64 μg/mL.

## Figures and Tables

**Table 1:** ^1^H and ^13^C NMR data of compounds **1** and **2**.

No.	Compound 1 (DMSO-*d*_6_)		Compound 2 (DMSO-*d*_6_)	
	δ_H_ (Mult, *J* in Hz) *^a^*	δ_C_, Type *^b^*	δ_H_ (Mult, *J* in Hz) *^a^*	δ_C_, Type *^c^*
1		172.1, C		180.2, C
2	3.79, ddd (10.1, 8.3, 5.1)	49.4, CH	3.67, m	51.0, CH
3	2.32, dd (14.1, 5.1) 2.24, dd (14.1, 10.1)	32.4, CH_2_	2.39, d (13.0) 2.14, dd (14.6,11.8)	33.4, CH_2_
4		56.1, C		56.3, C
5		129.5, C		130.0, C
6	6.57, d (7.7)	116.7, CH	6.57, d (7.7)	116.9, CH
7	6.79, t (7.7)	121.1, CH	6.75, t (7.7)	120.9, CH
8	6.70, d (7.7)	115.1, CH	6.65, d (7.7)	115.0, CH
9		141.0, C		141.0, C
10		130.5, C		130.5, C
12		179.3, C		180.2, C
13		41.8, C		41.7, C
14	6.04, dd (17.4, 10.9)	143.2, CH	6.03, dd (17.0, 11.0)	143.6, CH
15	5.08, d (10.9) 4.97, d (17.4)	113.5, CH_2_	5.02, d (11.0) 4.93, d (17.0)	113.1, CH_2_
16	1.02, s	21.7, CH_3_	1.01, s	21.8, CH_3_
17	0.94, s	21.4, CH_3_	0.93, s	21.6, CH_3_
1′		173.0, C		172.6, C
2′	3.90, ddd (9.6, 6.0, 3.1)	72.1, CH	3.76, d (10.5)	72.9, CH
3′	2.79, dd (13.9, 3.1) 2.61, dd (13.9, 9.6)	39.9, CH_2_	2.77, d (13.5) 2.61, dd (13.5, 10.5)	40.1, CH_2_
4′		138.9, C		140.0, C
5′/9′	7.22, d (7.2)	129.4, CH	7.26, m	129.4, CH
6′/8′	7.26, t (7.2)	127.8, CH	7.26, m	127.9, CH
7′	7.18, t (7.2)	125.8, CH	7.18, m	125.7, CH
1-OMe	3.38, s	51.7, CH_3_		
9-OH	9.39, s		9.61, br s	
11-NH	10.15, s		10.05, br s	
2′-OH	5.52, d (6.0)		5.45, br s	
10′-NH	7.35, d (8.3)		6.86, br s	

*^a^* Measured at 500 MHz; *^b^* Measured at 125 MHz; *^c^* Measured at 150 MHz.

**Table 2:** ^1^H (500 MHz) and ^13^C NMR (125 MHz) data of compound **3** in DMSO-*d*_6_.

No.	δ_H_ (Mult, *J* in Hz)	δ_C_, Type	No.	δ_H_ (Mult, *J* in Hz)	δ_C_, Type
1	2.47, m 2.27, m	33.3, CH_2_	17	1.89, m	52.7, CH
2	1.89, m 1.76, m	33.7, CH_2_	18	0.82, s	15.5, CH_3_
3		197.7, C	19	0.95, s	16.6, CH_3_
4	5.66, s	122.7, CH	20	2.90, m	35.4, CH
5		162.9, C	21	1.03, d (7.2)	23.1, CH_3_
6	6.10, d (9.7)	124.4, CH	22	5.37, dd (15.2, 9.1)	137.9, CH
7	7.17, d (9.7)	134.4, CH	23	5.18, dd (15.2, 9.4)	127.1, CH
8		127.5, C	24	2.20, td (9.4, 3.7)	52.9, CH
9	2.31, m	44.8, CH	25		69.9, C
10		36.2, C	26	1.09, s	29.5, CH_3_
11	1.76, m 1.53, m	28.1, CH_2_	27	1.01, s	26.4, CH_3_
12	3.50, d (9.8)	74.3, CH	28	4.26, dd (10.5, 3.7) 3.96, t (10.5)	64.2, CH_2_
13		48.7, C	29		170.3, C
14		156.3, C	30	1.93, s	20.7, CH_3_
15	4.70, m	68.1, CH	12-OH	4.70, br s	
16	1.52, m 1.60, m	35.4, CH_2_	15-OH	4.80, br s	

**Table 3:** Antibacterial activity of compounds **1**–**4** (MIC, μg/mL).

	1	2	3	4	Chloramphenicol
EC	64	16	–	32	1
EI	32	64	32	64	0.5
ML	32	64	–	32	2
VS	64	–	64	32	0.5

EC: *E. coli*. EI: *Ed. ictaluri*. ML: *M. luteus*. VS: *V. alginolyticus*.

## Supplementary Materials

The following are available online at http://www.mdpi.com/1660-3397/16/4/114/s1, 1D and 2D NMR spectra and ECDs of compounds **1**–**3** as well as crystal packing of compound **3**.
