# Supplementary material for: Two New Diketomorpholine Derivatives and a New Highly Conjugated Ergostane-Type Steroid from the Marine Algal-Derived Endophytic Fungus Aspergillus alabamensis EN-547
Source: Mar Drugs. 2018 Mar 31;16(4):114. doi: 10.3390/md16040114 (PMC5923401; doi:10.3390/md16040114)

Supplementary Material

Two New Diketomorpholine Derivatives and a New Highly Conjugated Ergostane-Type Steroid from the Marine Algal-Derived Endophytic Fungus, *Aspergillus alabamensis* EN-547

Sui-Qun Yang [1,2], Xiao-Ming Li[1], Xin Li [1], Lu-Ping Chi [1,2], and Bin-Gui Wang [1,*]

[1] Key Laboratory of Experimental Marine Biology, Institute of Oceanology, Chinese Academy of Sciences and Laboratory of Marine Biology and Biotechnology, Qingdao National Laboratory for Marine Science and Technology, Nanhai Road 7, Qingdao 266071, China; E-Mails: suiqunyang@163.com (S.-Q.Y.); lixmqdio@126.com (X.-M.L.); lixin871014@163.com (X.L.); 1061825010@qq.com (L.-P.C.)

[2] University of Chinese Academy of Sciences, Yuquan Road 19A, Beijing 100049, China

* Correspondence: wangbg@ms.qdio.ac.cn; Tel.: +86-532-82898553.

Table of Contents

Figure S1. HPLC analysis of the original EtOAc extract of *A. alabamensis* EN-547;

Figure S2. ^{1}H NMR (500 MHz, DMSO-d_6) spectrum of compound **1**;

Figure S3. ^{13}C NMR (125 MHz, DMSO-d_6) and DEPT spectra of compound **1**;

Figure S4. COSY spectrum of compound **1**;

Figure S5. HMBC spectrum of compound **1**;

Figure S6. NOESY spectrum of compound **1**;

Figure S7. HRESIMS spectrum of compound **1**;

Figure S8. HPLC analysis of compound **1**;

Figure S9. ECD spectrum of compound **1**;

Figure S10. ^{1}H NMR (500 MHz, DMSO-d_6) spectrum of compound **2**;

Figure S11. ^{13}C NMR (150 MHz, DMSO-d_6) spectrum of compound **2**;

Figure S12. HSQC spectrum of compound **2**;

Figure S13. COSY spectrum of compound **2**;

Figure S14. HMBC spectrum of compound **2**;

Figure S15. NOESY spectrum of compound **2**;

Figure S16. HRESIMS spectrum of compound **2**;

Figure S17. HPLC analysis of compound **2**;

Figure S18. ECD spectrum of compound **2**;

Figure S19. ^{1}H NMR (500 MHz, DMSO-d_6) spectrum of compound **3**;

Figure S20. ^{13}C NMR (125 MHz, DMSO-d_6) and DEPT spectra of compound **3**;

Figure S21. COSY spectrum of compound **3**;

Figure S22. HMBC spectrum of compound **3**;

Figure S23. NOESY spectrum of compound **3**;

Figure S24. HRESIMS spectrum of compound **3**;

Figure S25. HPLC analysis of compound **3**;

Figure S26. ECD spectrum of compound **3**;

Figure S27. Crystal packing of compound **3** at 293(2) K.

Figure S1. HPLC analysis of the original EtOAc extract of *A. alabamensis* EN-547

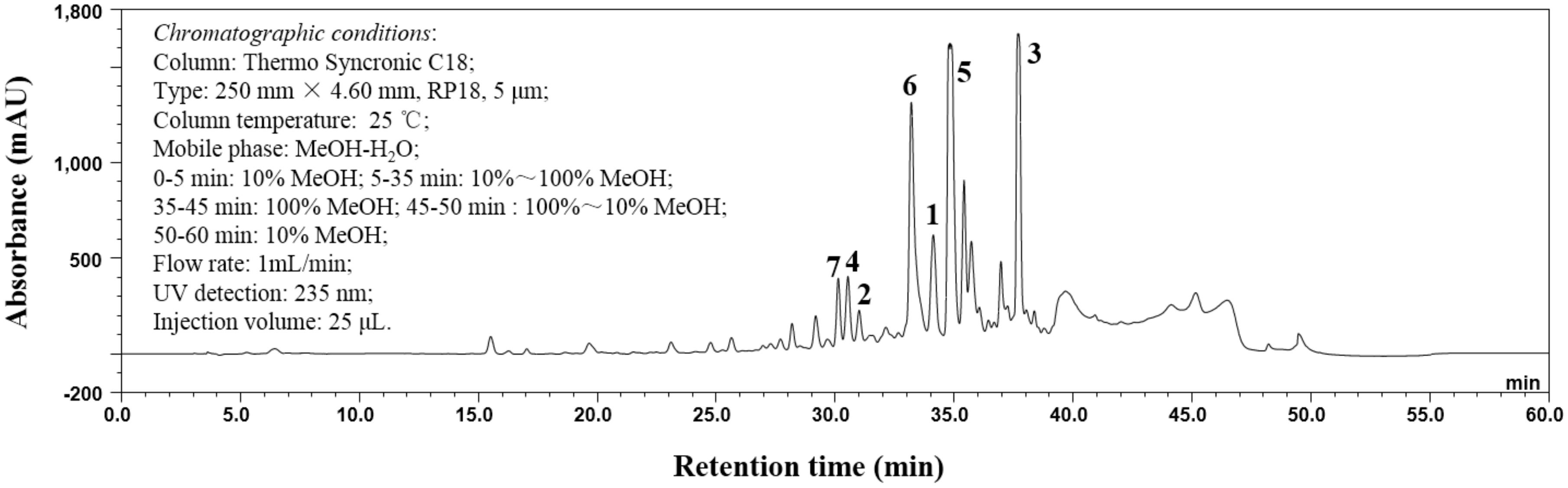

Figure S2. ^{1}H NMR (500 MHz, DMSO-d_6) spectrum of compound **1**

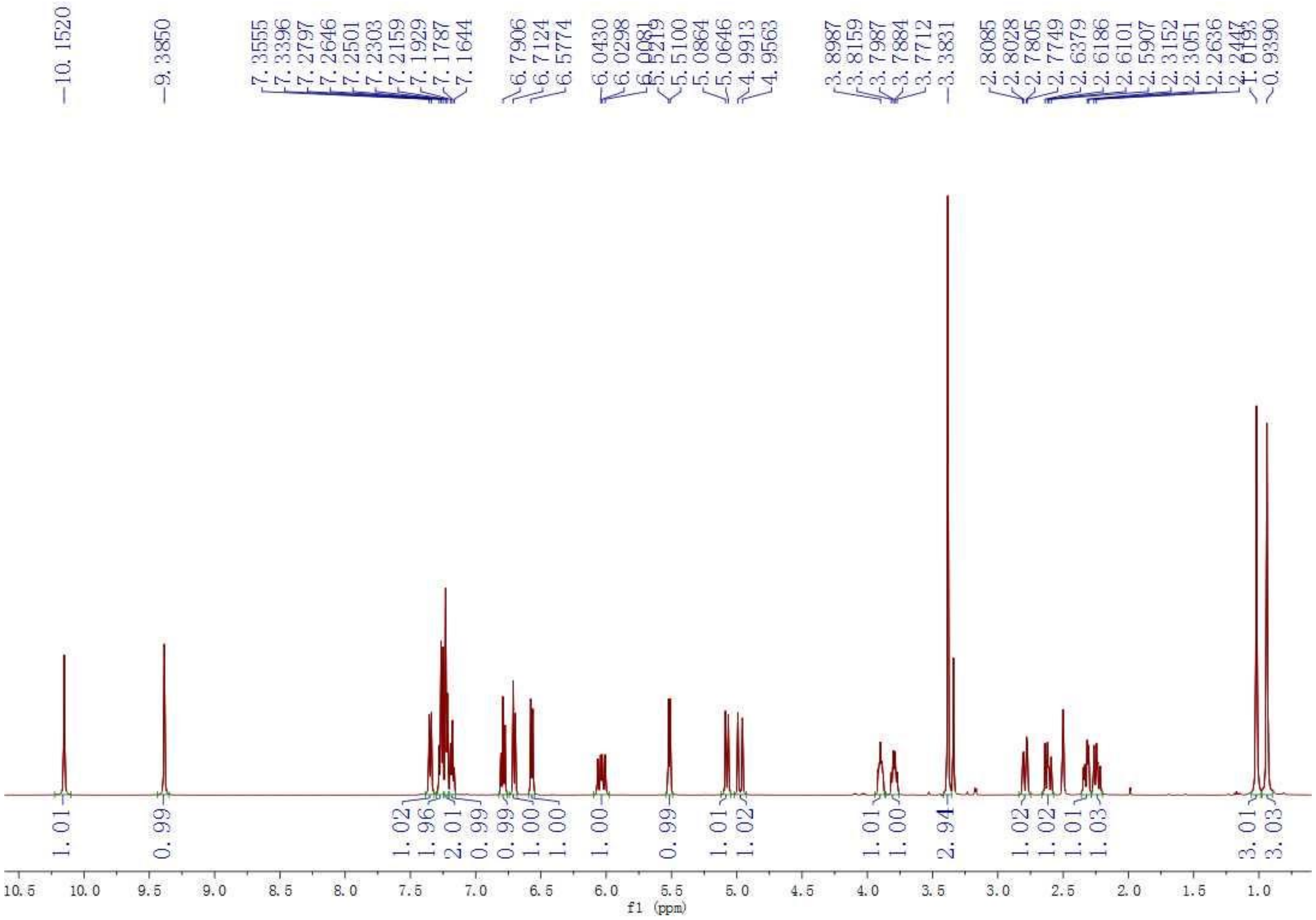

Figure S3. ^{13}C NMR (125 MHz, DMSO-d_6) and DEPT spectra of compound **1**

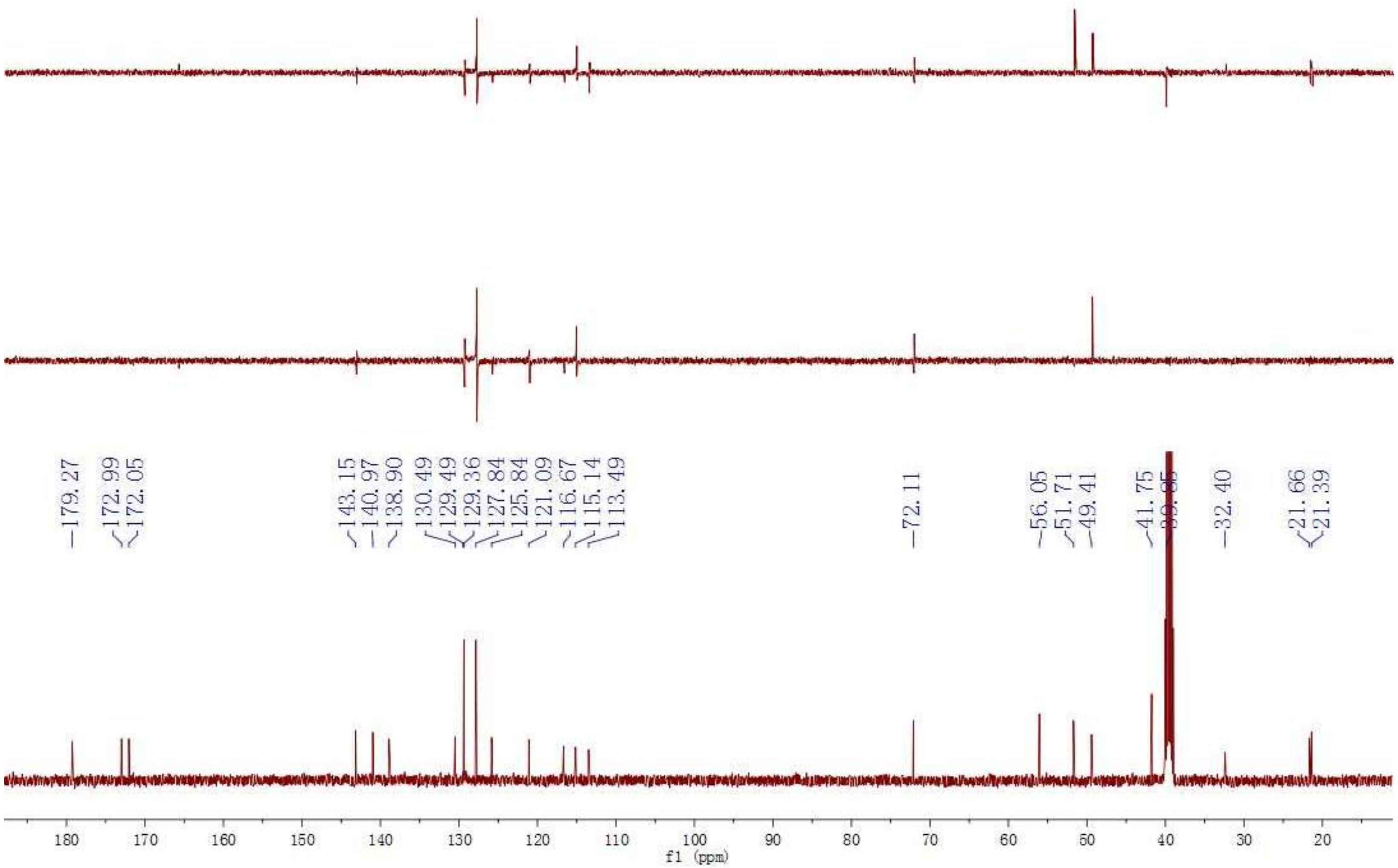

Figure S4. ^{1}H-^{1}H COSY spectrum of compound **1**

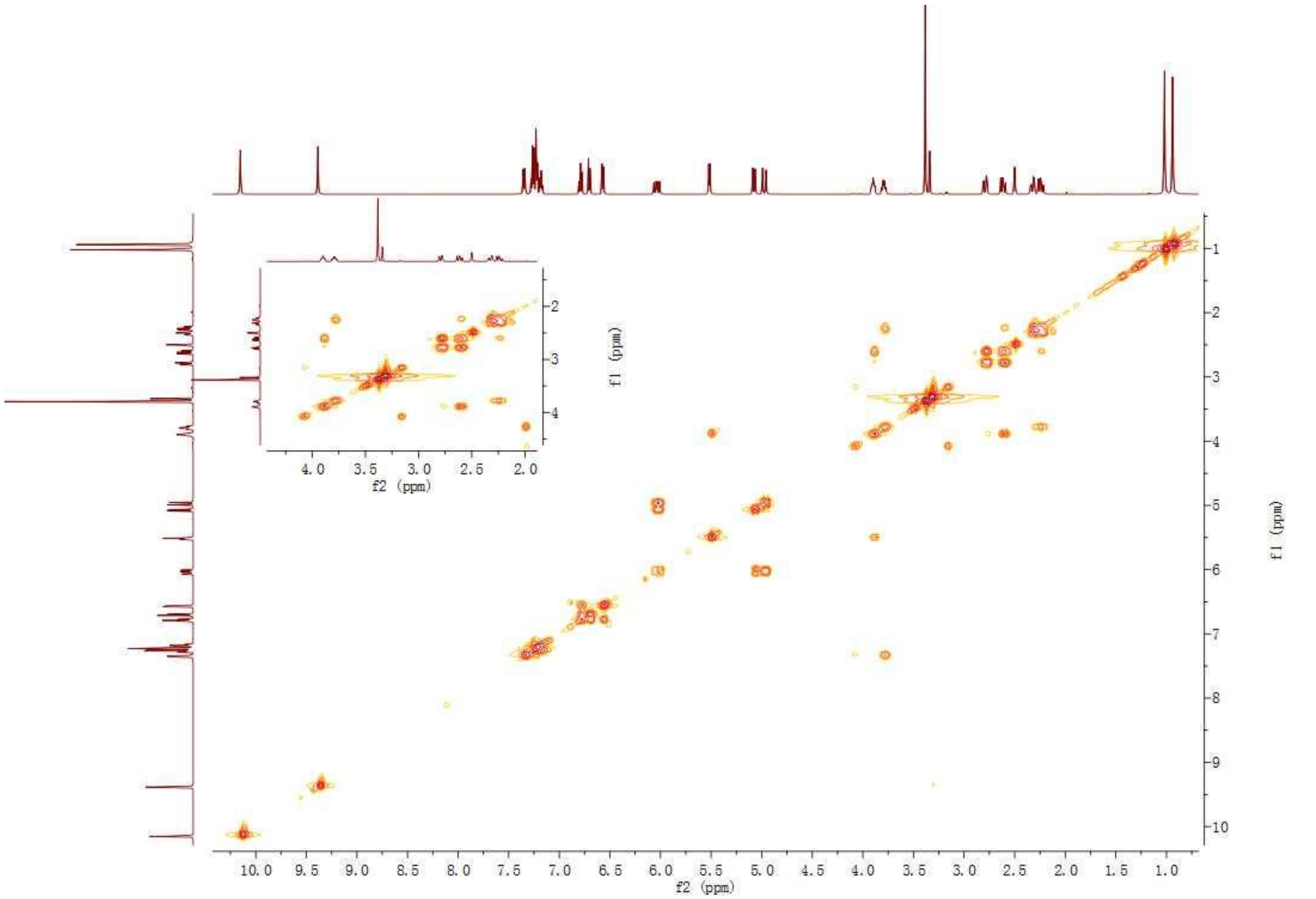

Figure S5. HMBC spectrum of compound **1**

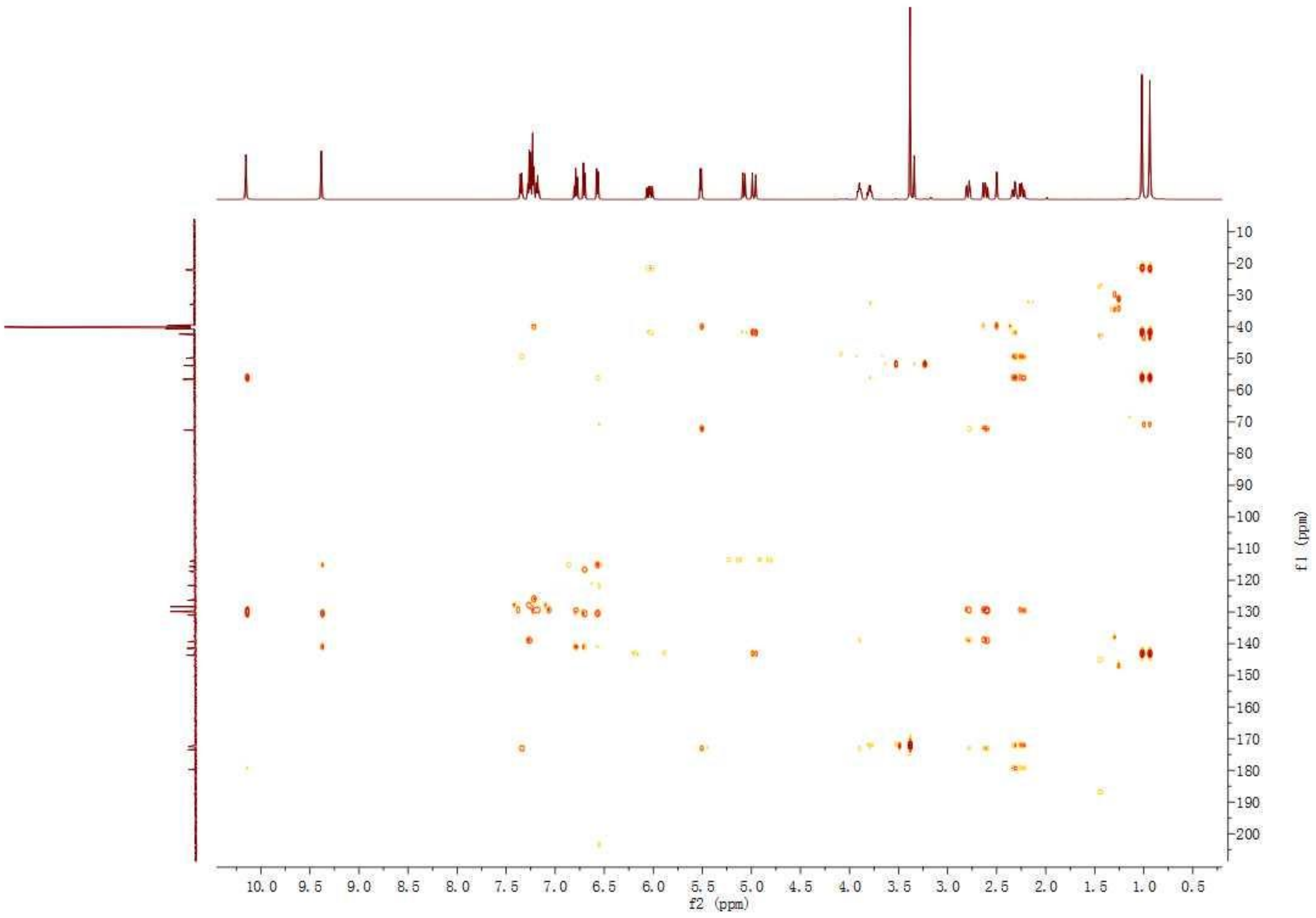

Figure S6. NOESY spectrum of compound **1**

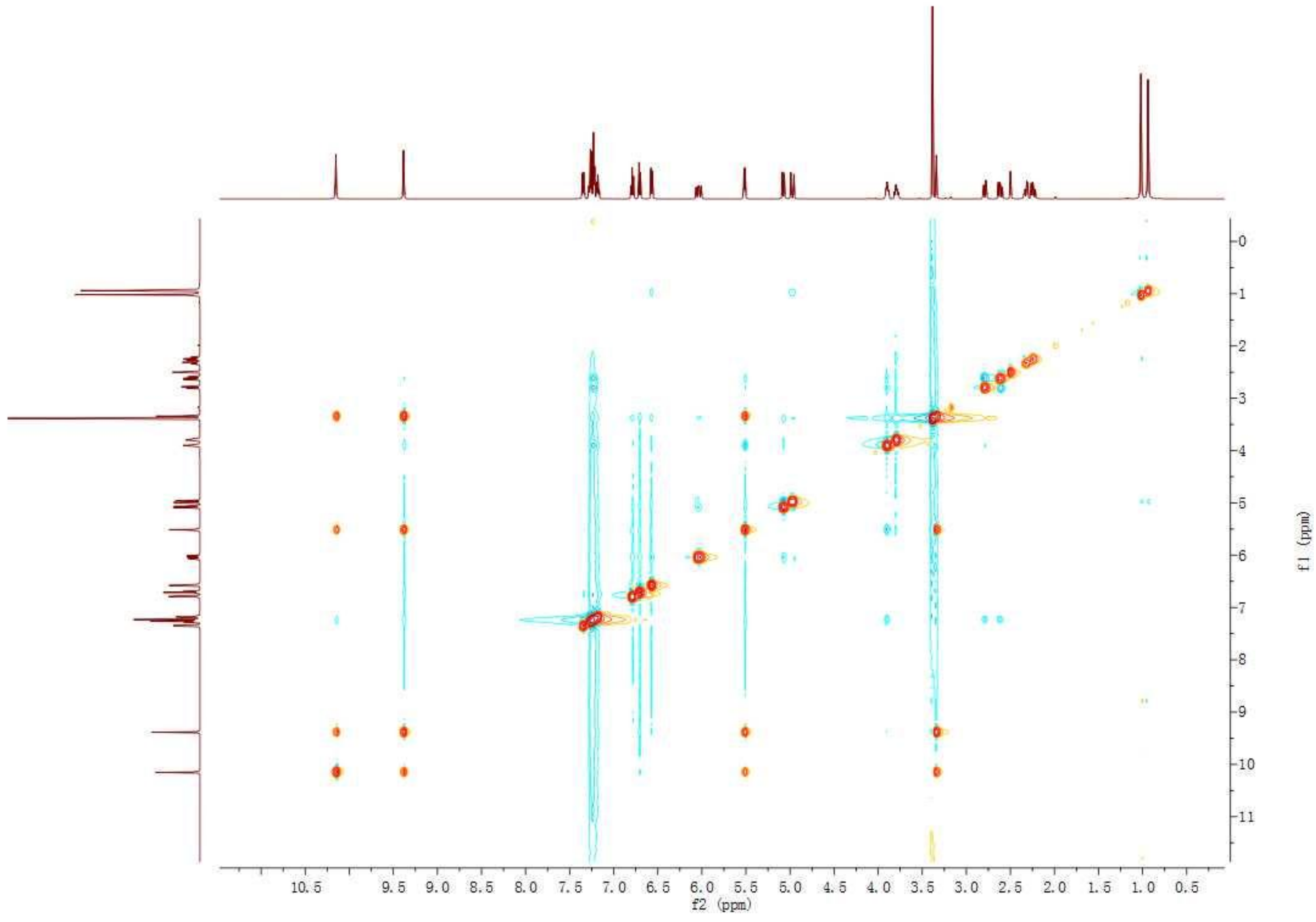

Figure S7. HRESIMS spectrum of compound **1**

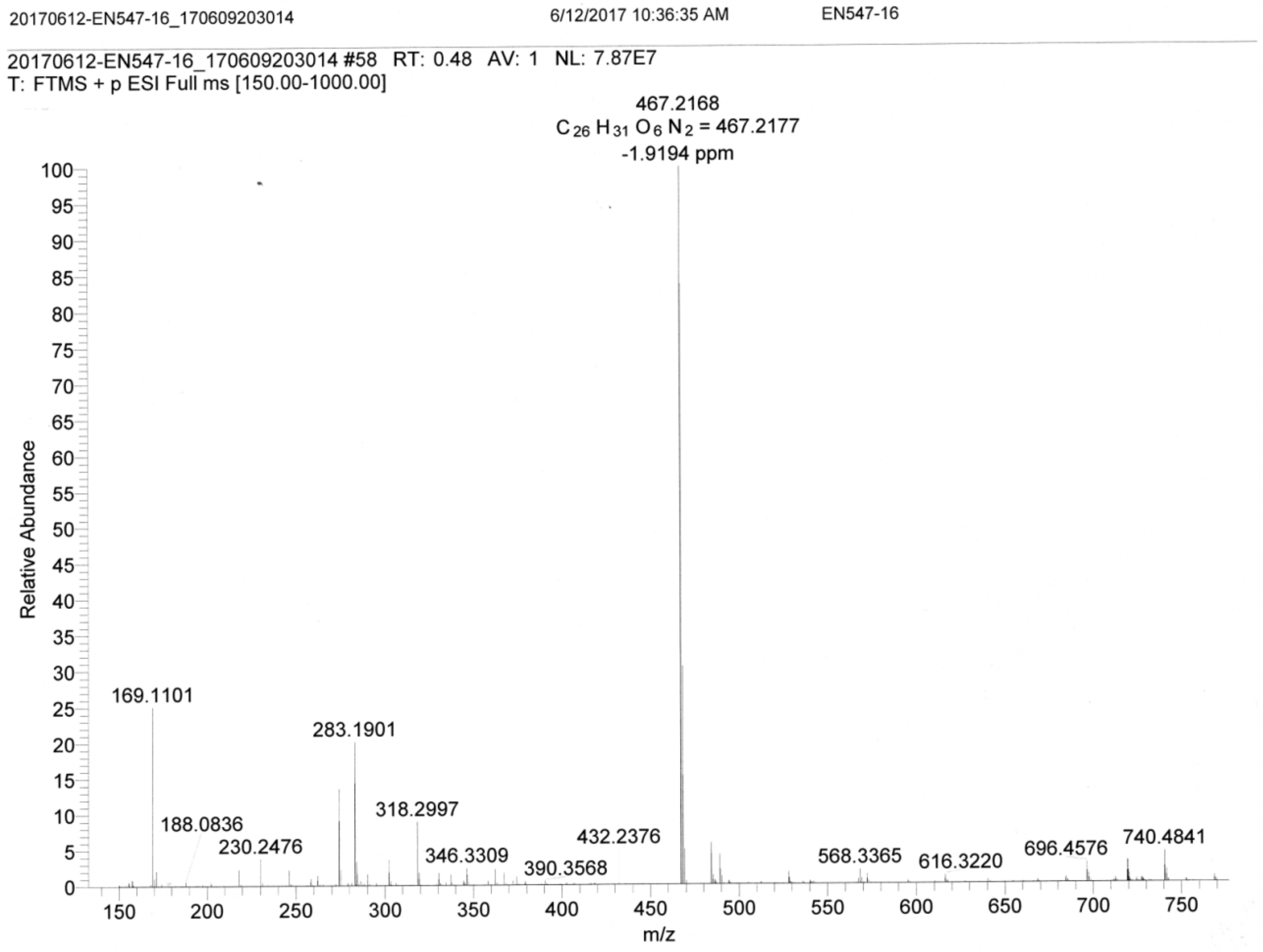

Figure S8. HPLC analysis of compound **1**

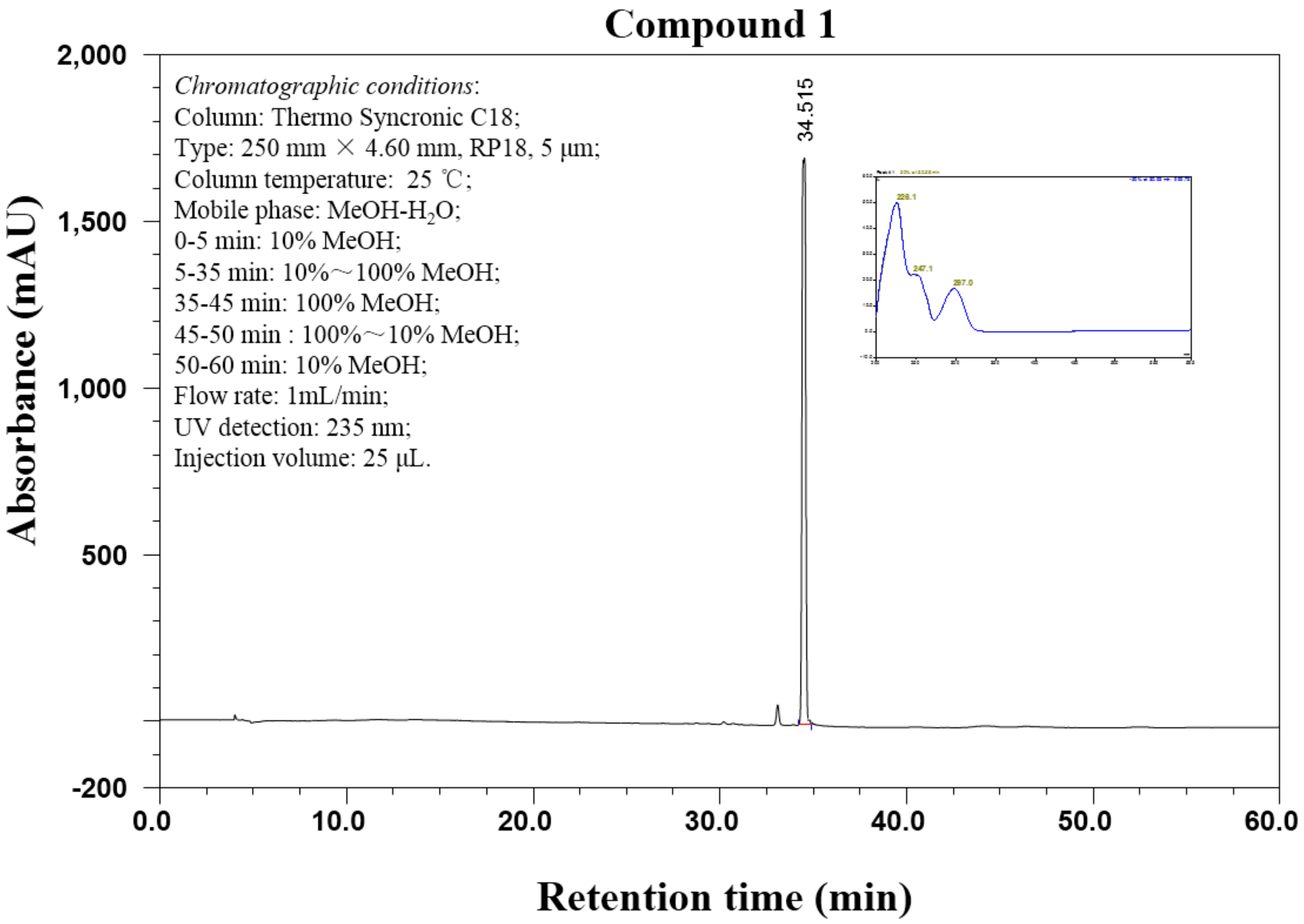

Figure S9. ECD spectrum of compound **1**

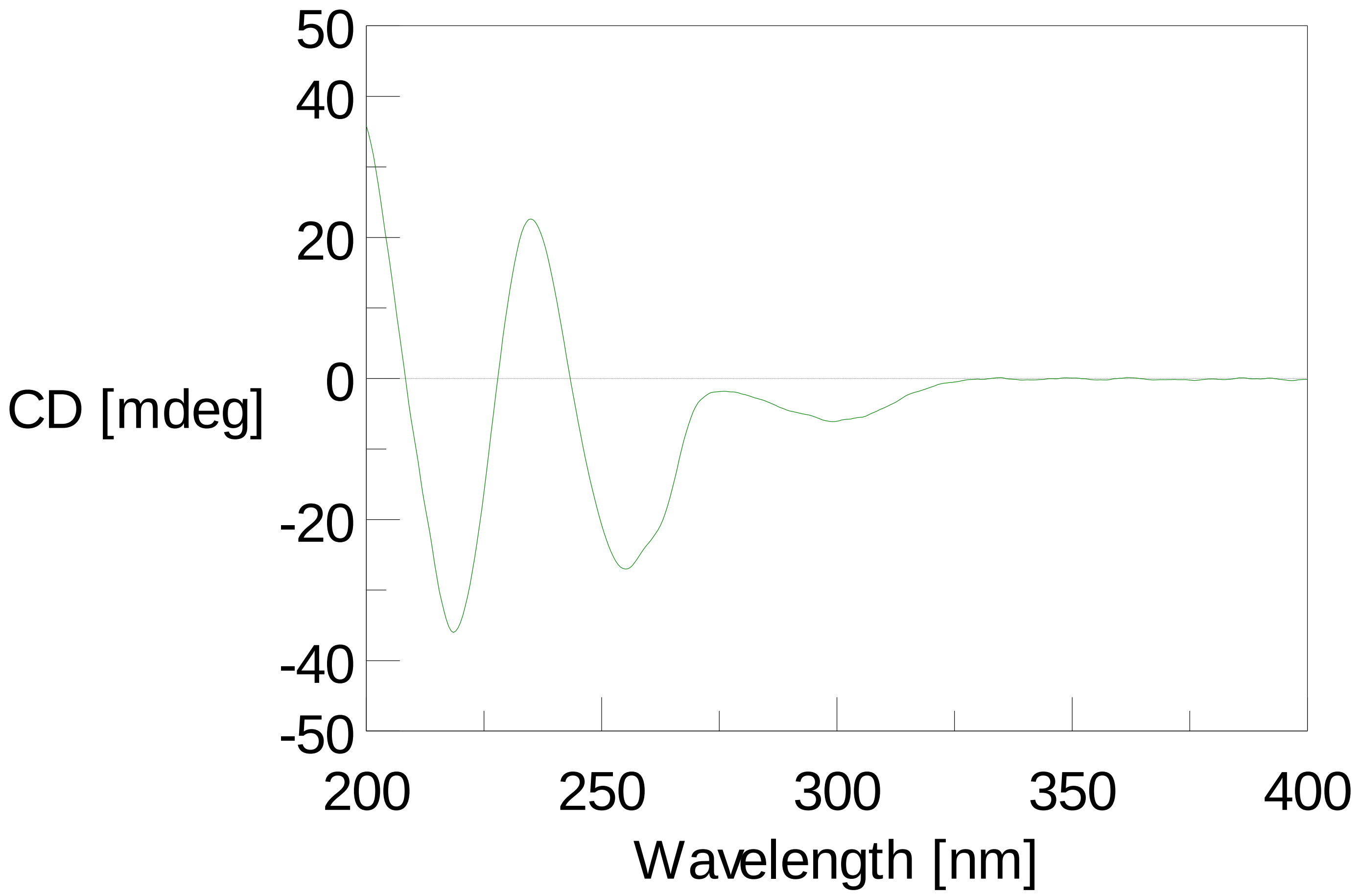

Figure S10. ^{1}H NMR (500 MHz, DMSO-d_6) spectrum of compound **2**

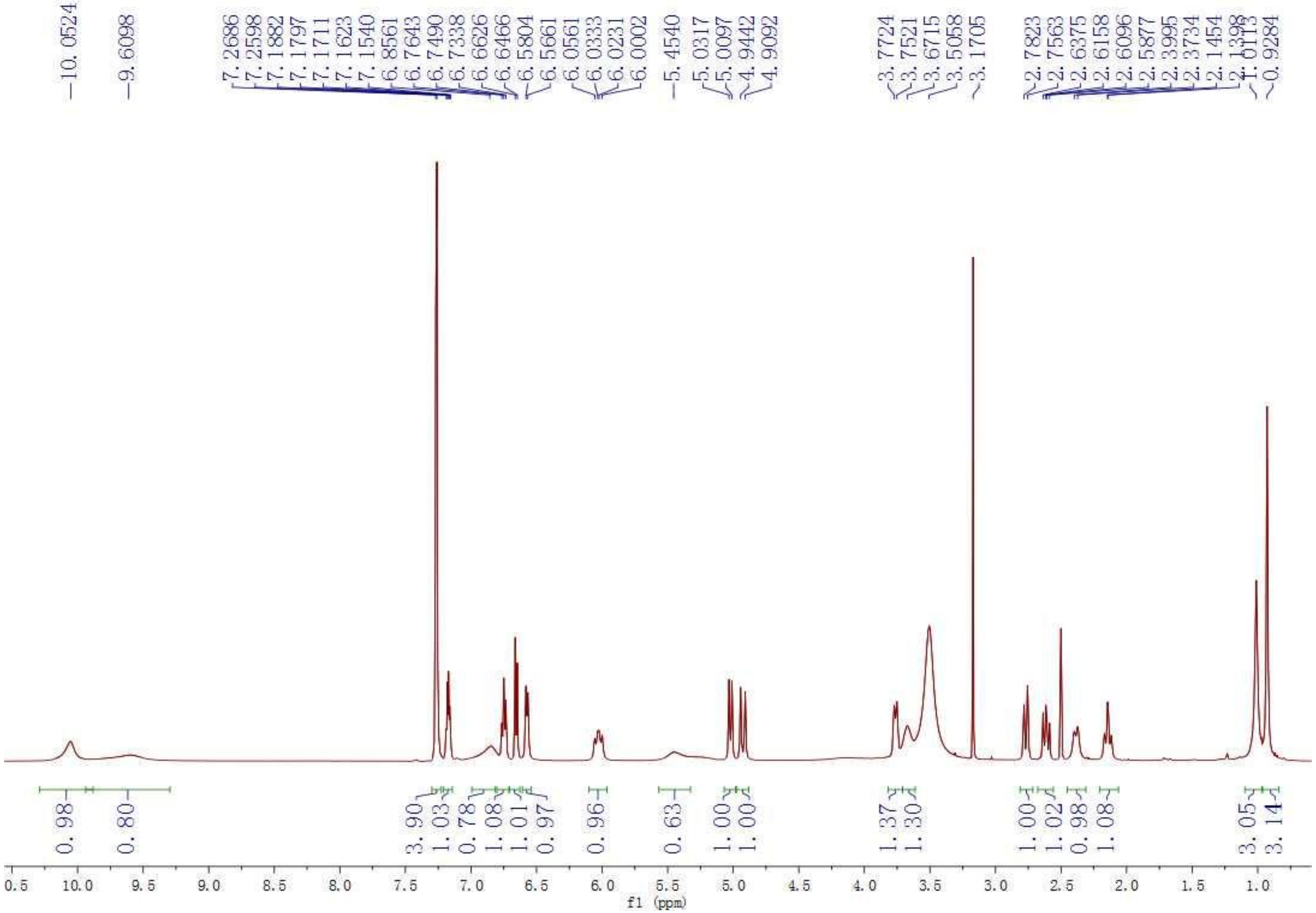

Figure S11. ^{13}C NMR (150 MHz, DMSO-d_6) spectrum of compound **2**

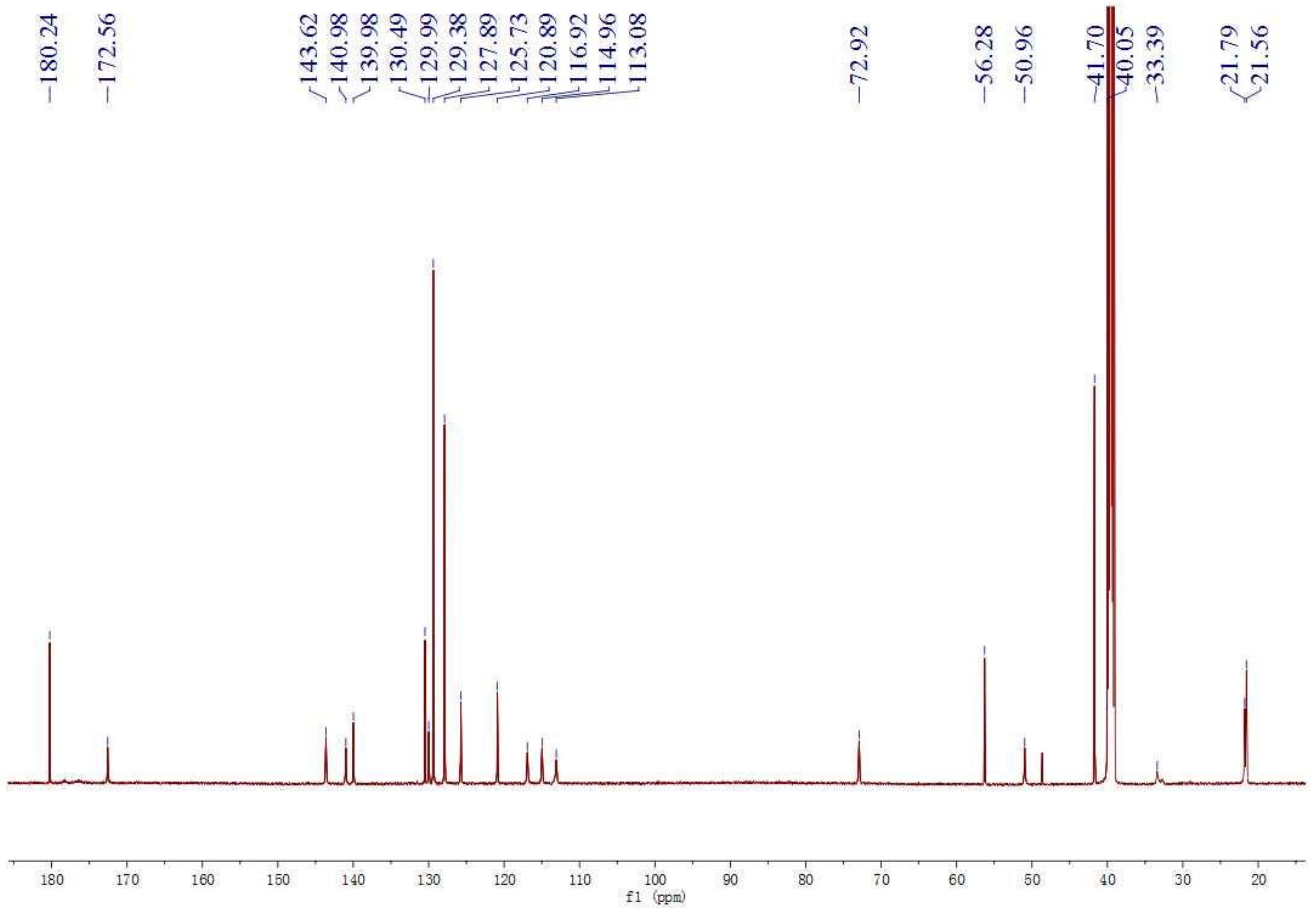

Figure S12. HSQC spectrum of compound **2**

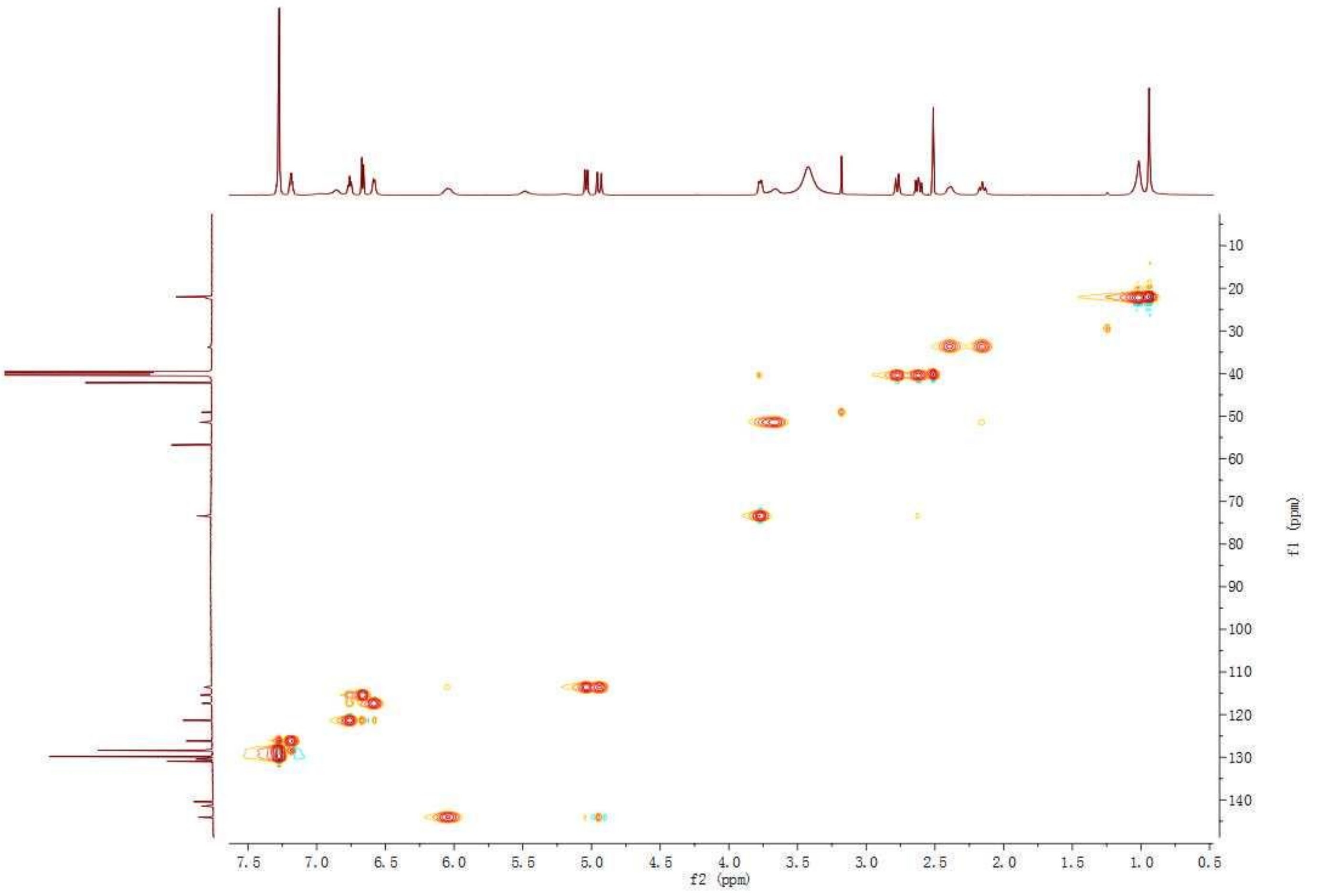

Figure S13. ^{1}H-^{1}H COSY spectrum of compound **2**

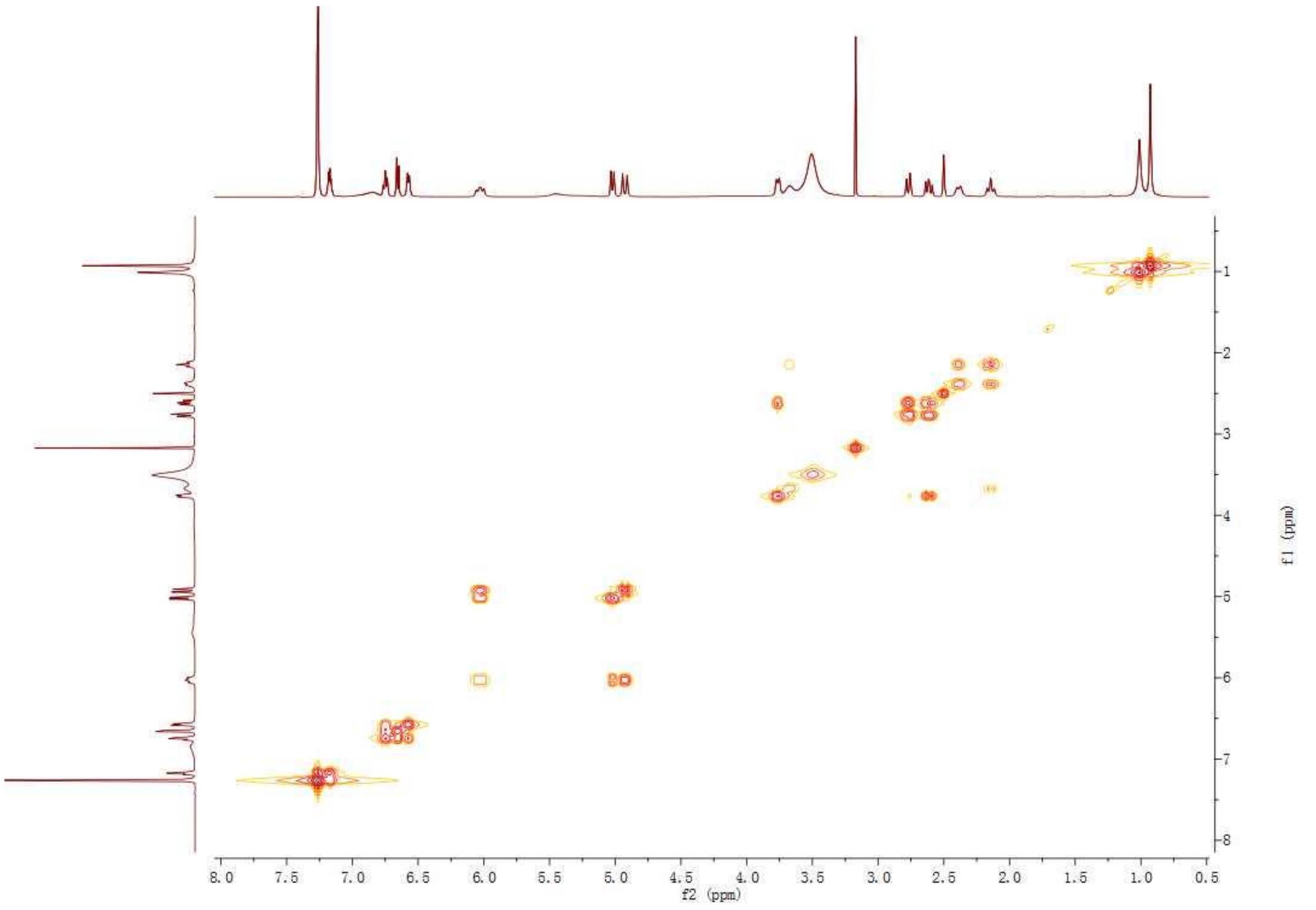

Figure S14. HMBC spectrum of compound **2**

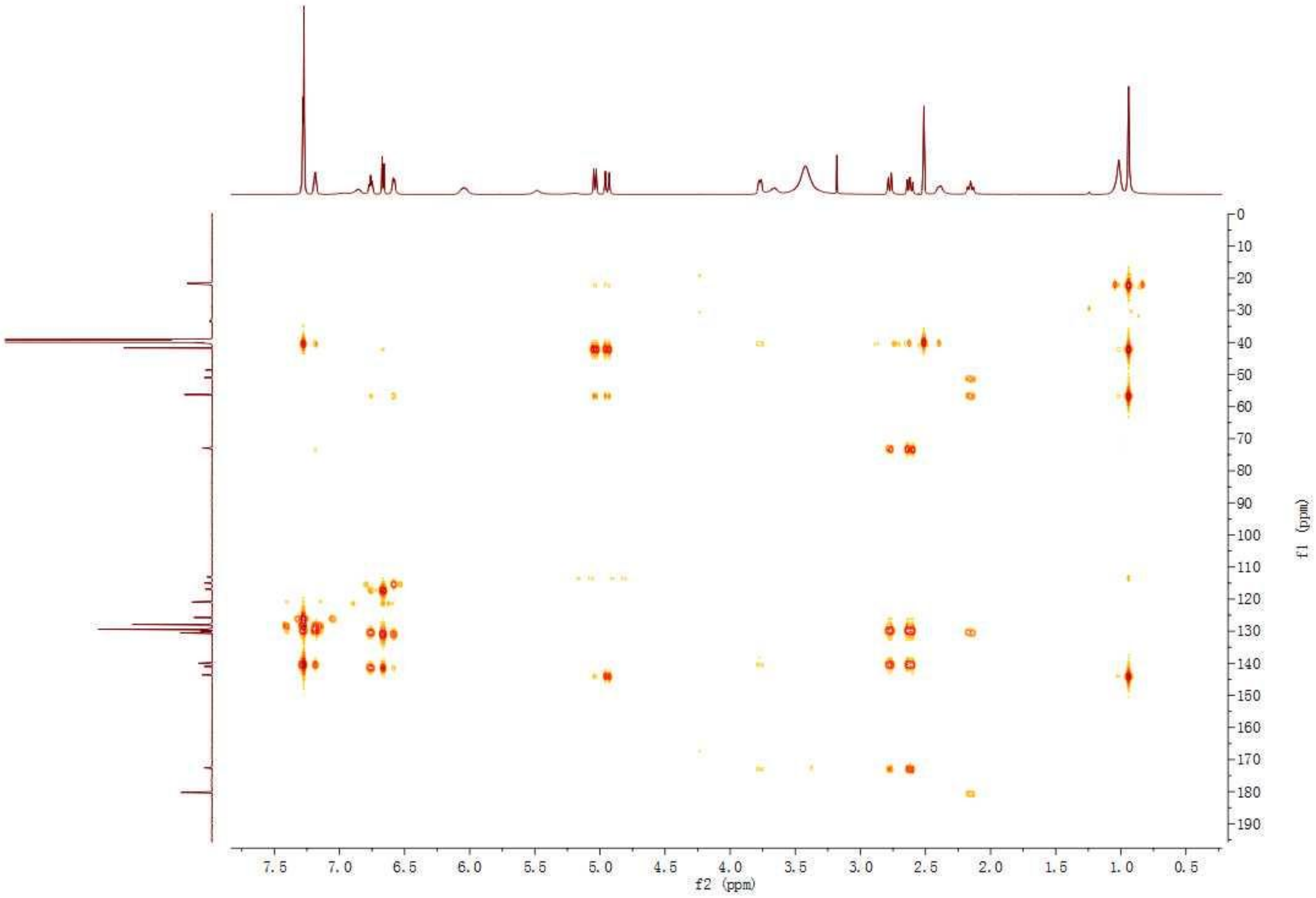

Figure S15. NOESY spectrum of compound **2**

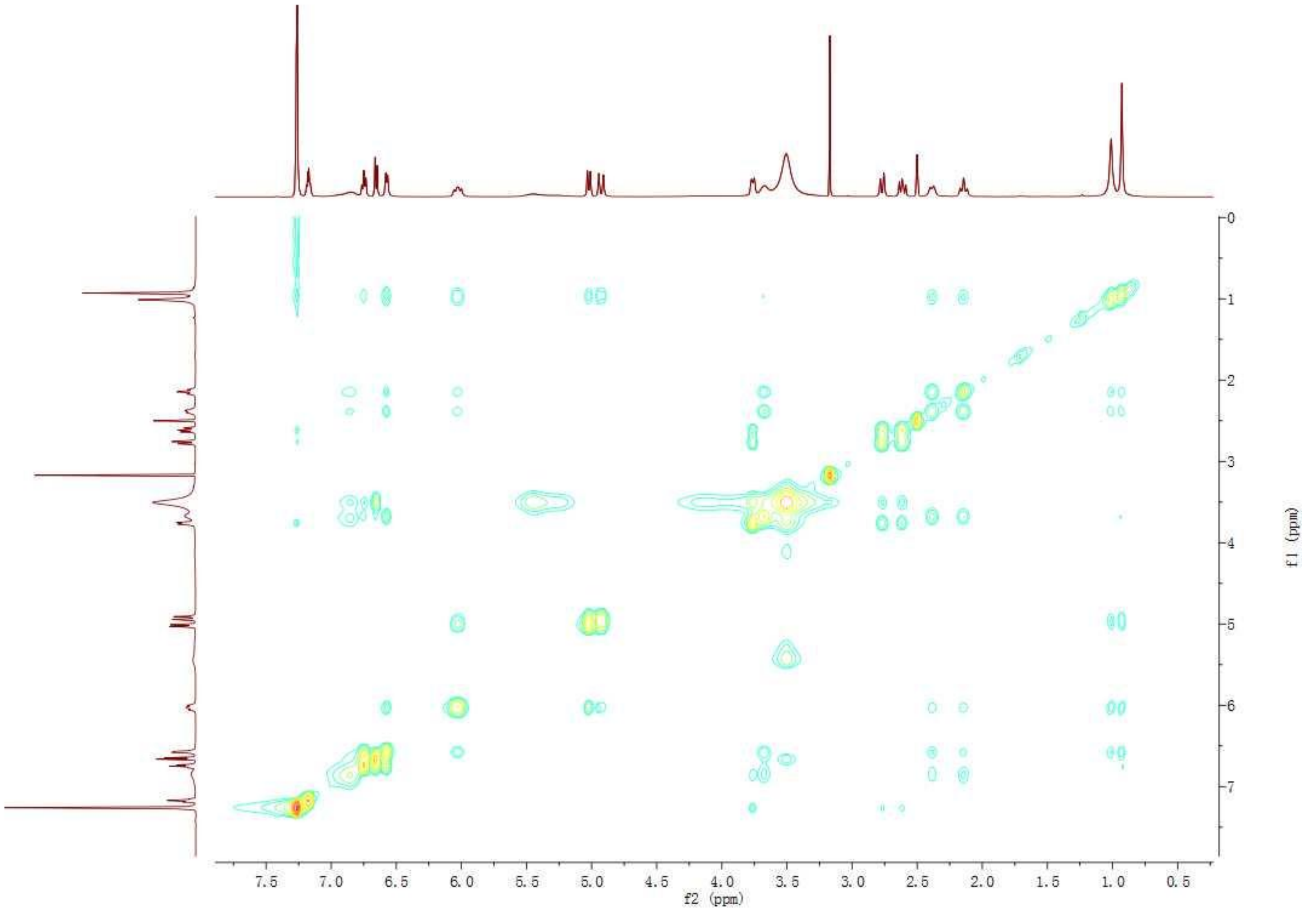

Figure S16. HRESIMS spectrum of compound **2**

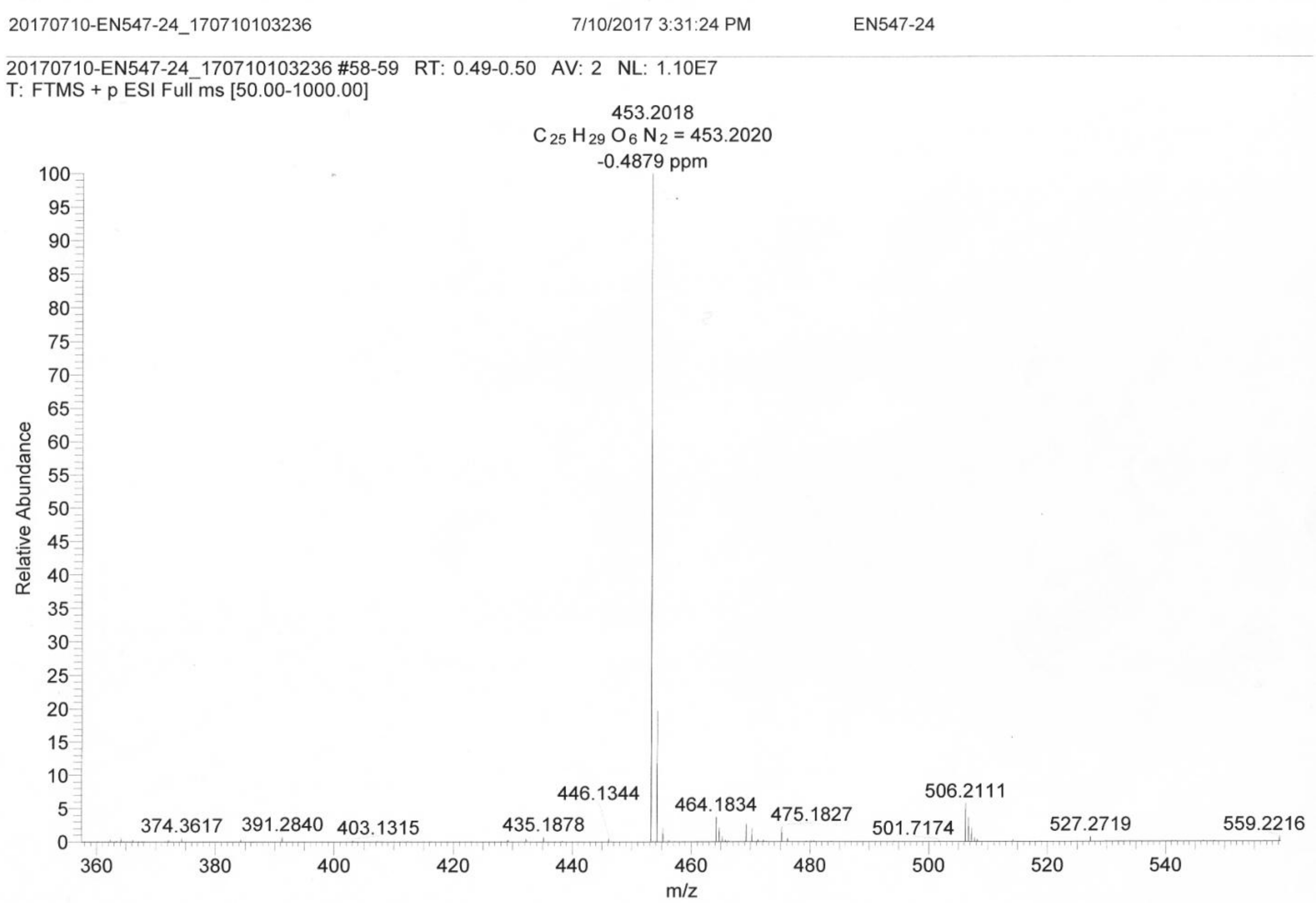

Figure S17. HPLC analysis of compound **2**

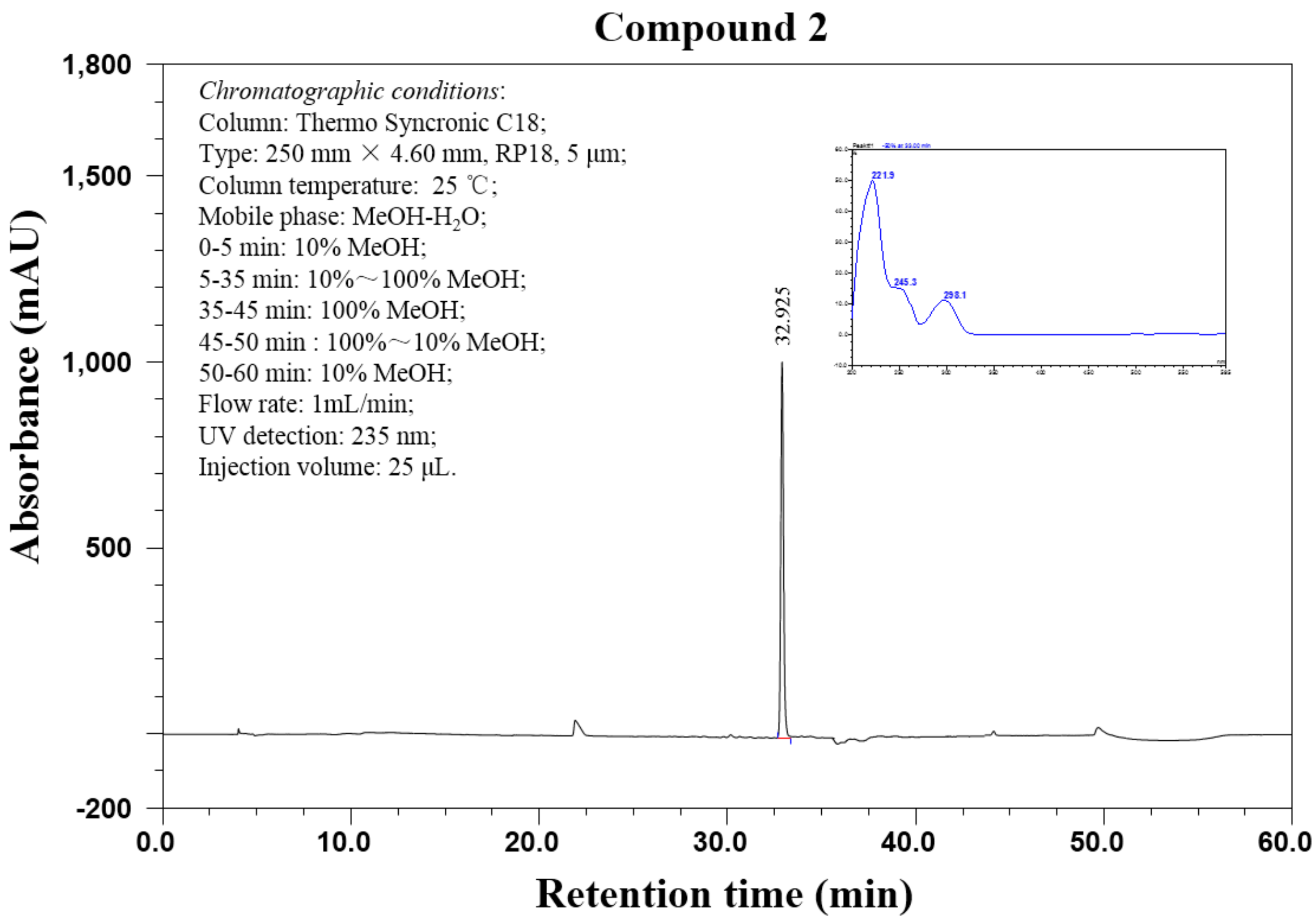

Figure S18. ECD spectrum of compound **2**

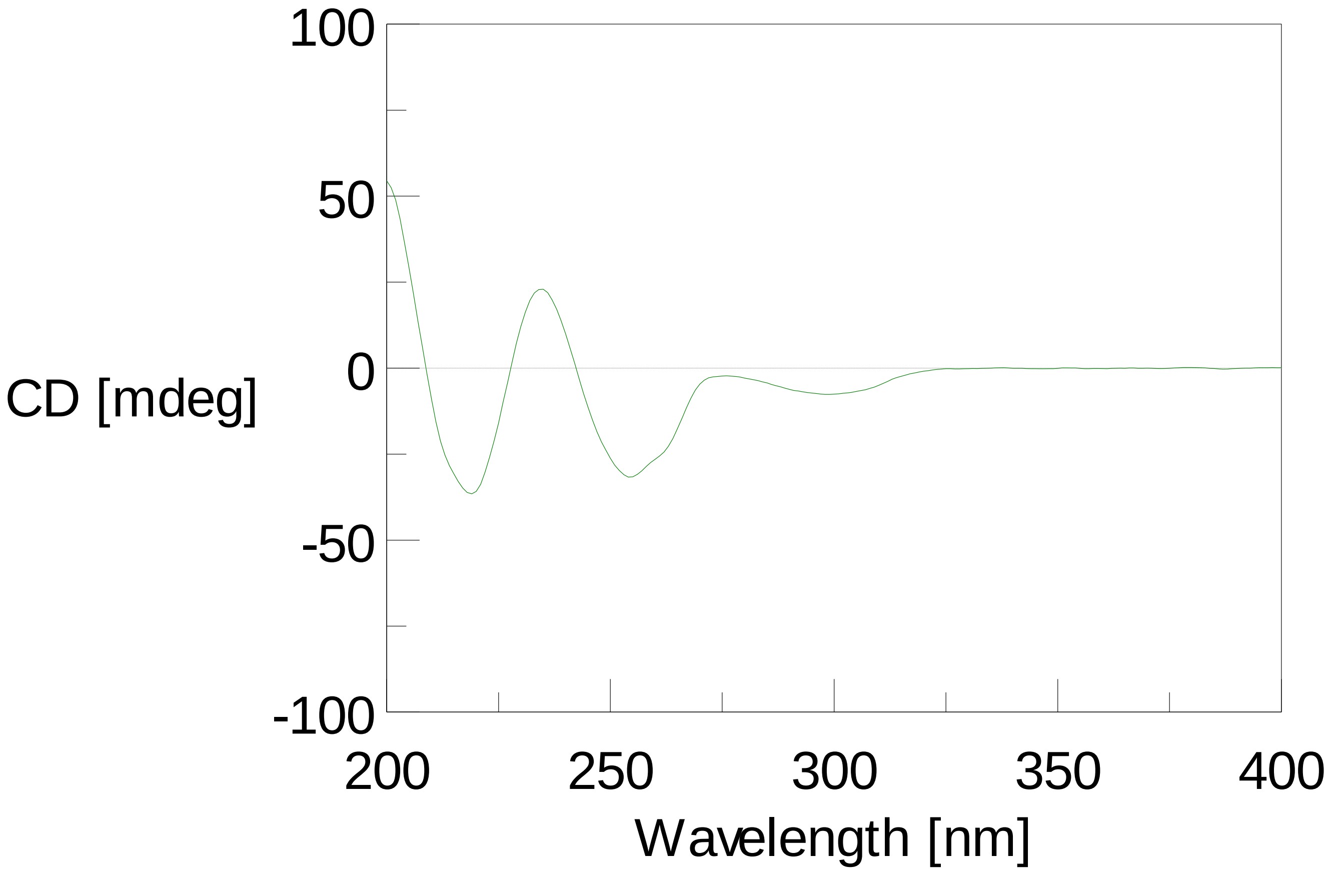

Figure S19. ^{1}H NMR (500 MHz, DMSO-d_6) spectrum of compound **3**

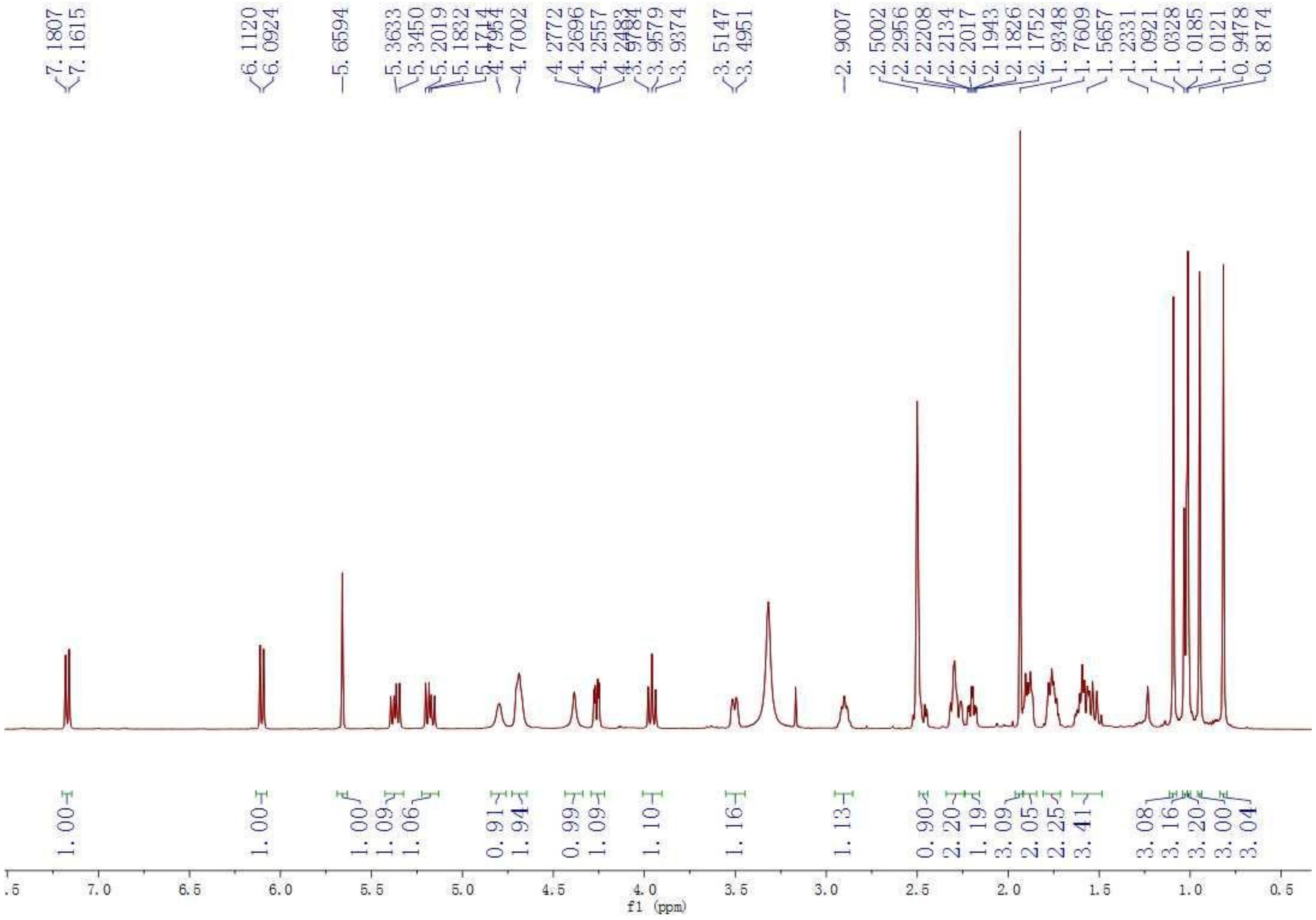

Figure S20. ^{13}C NMR (125 MHz, DMSO-d_6) and DEPT spectra of compound **3**

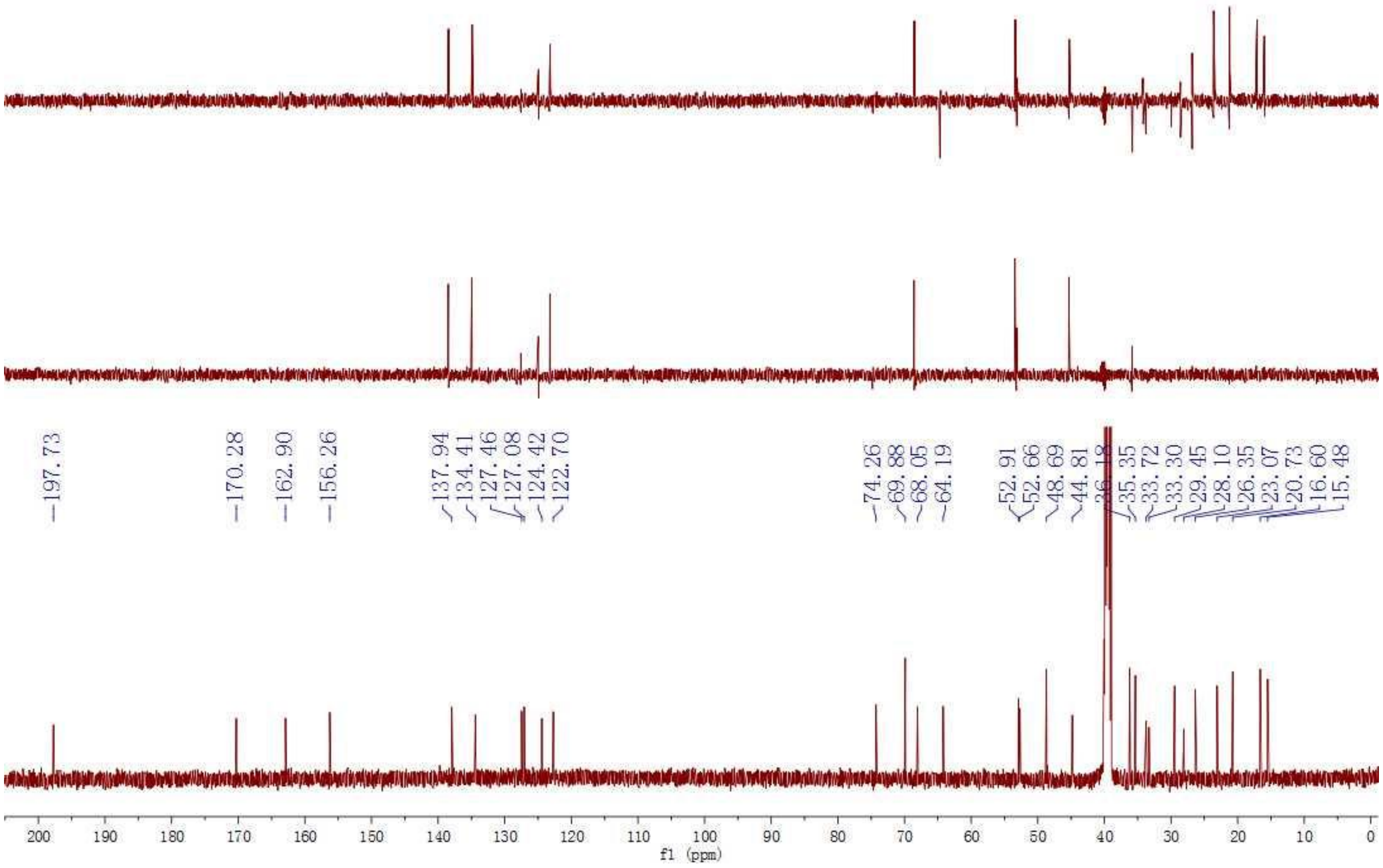

Figure S21. COSY spectrum of compound **3**

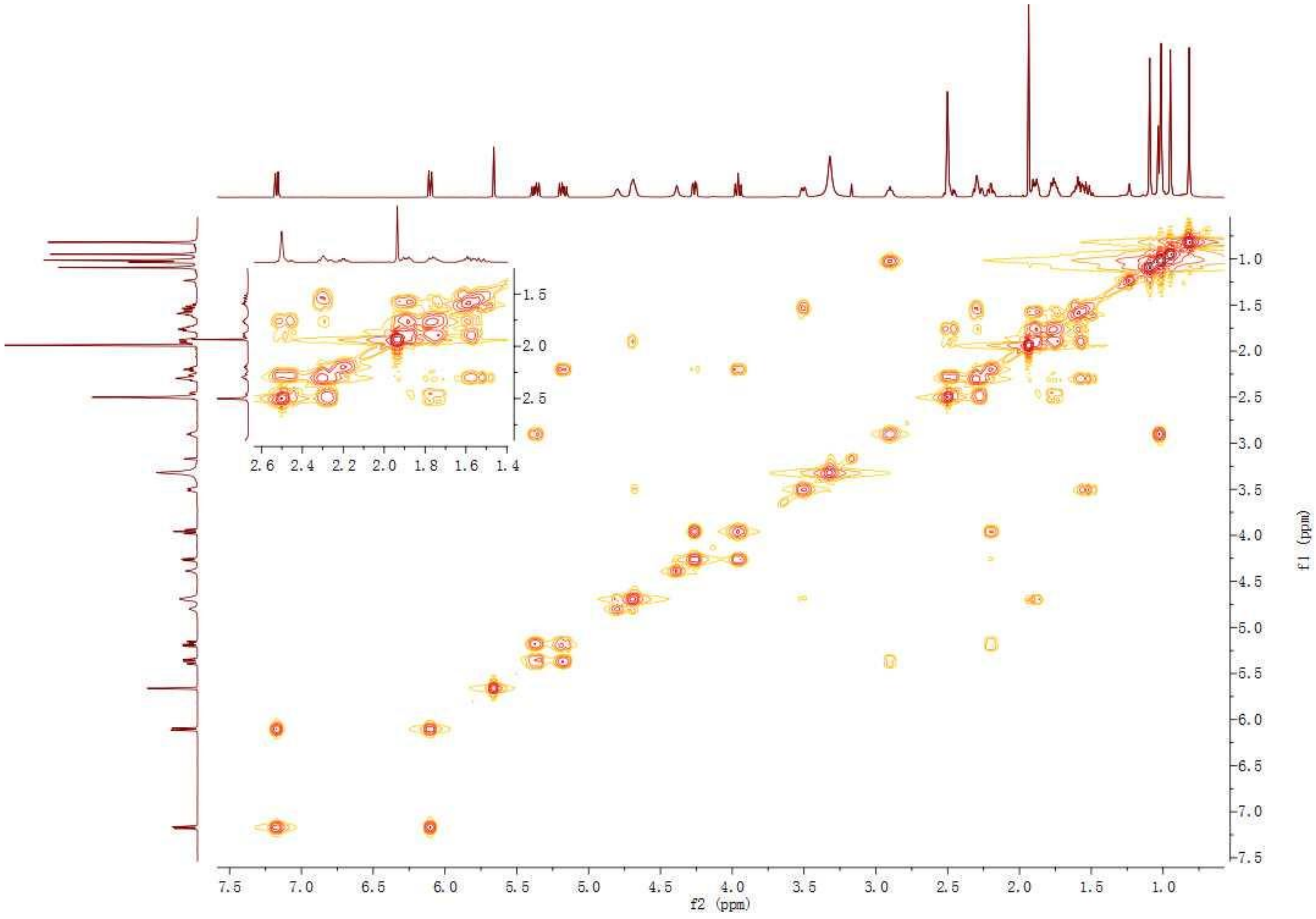

Figure S22. HMBC spectrum of compound **3**

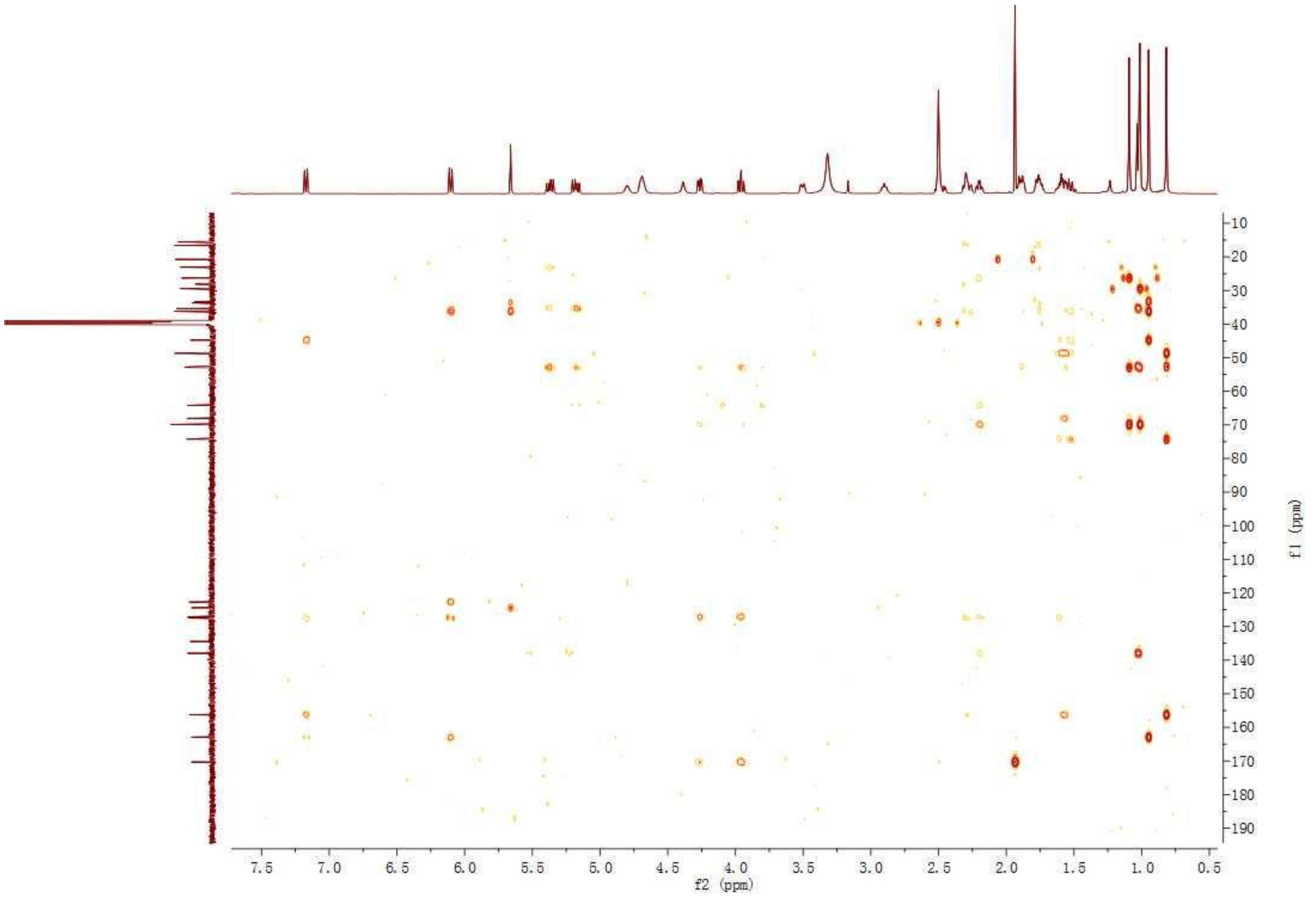

Figure S23. NOESY spectrum of compound **3**

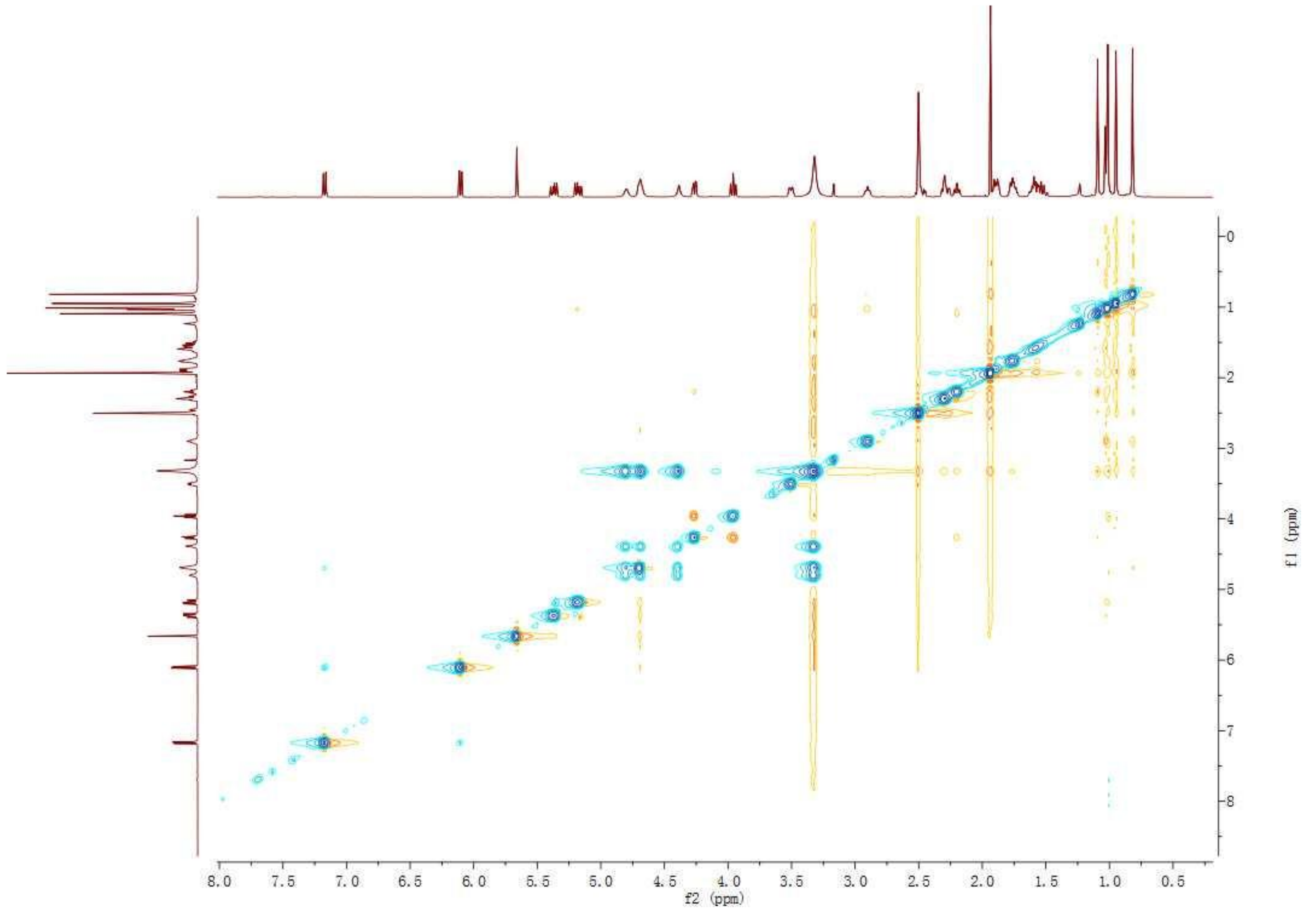

Figure S24. HRESIMS spectrum of compound **3**

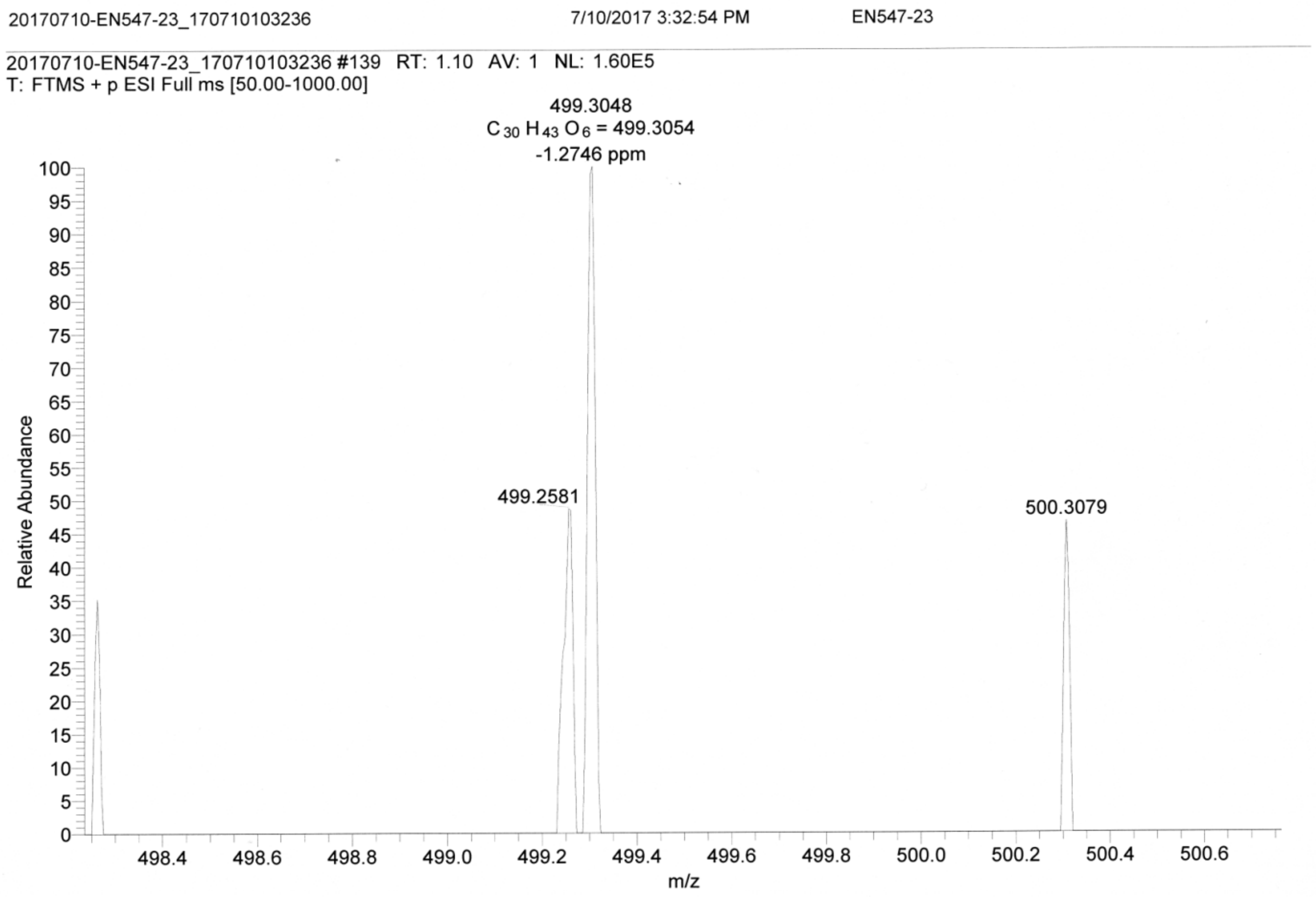

Figure S25. HPLC analysis of compound **3**

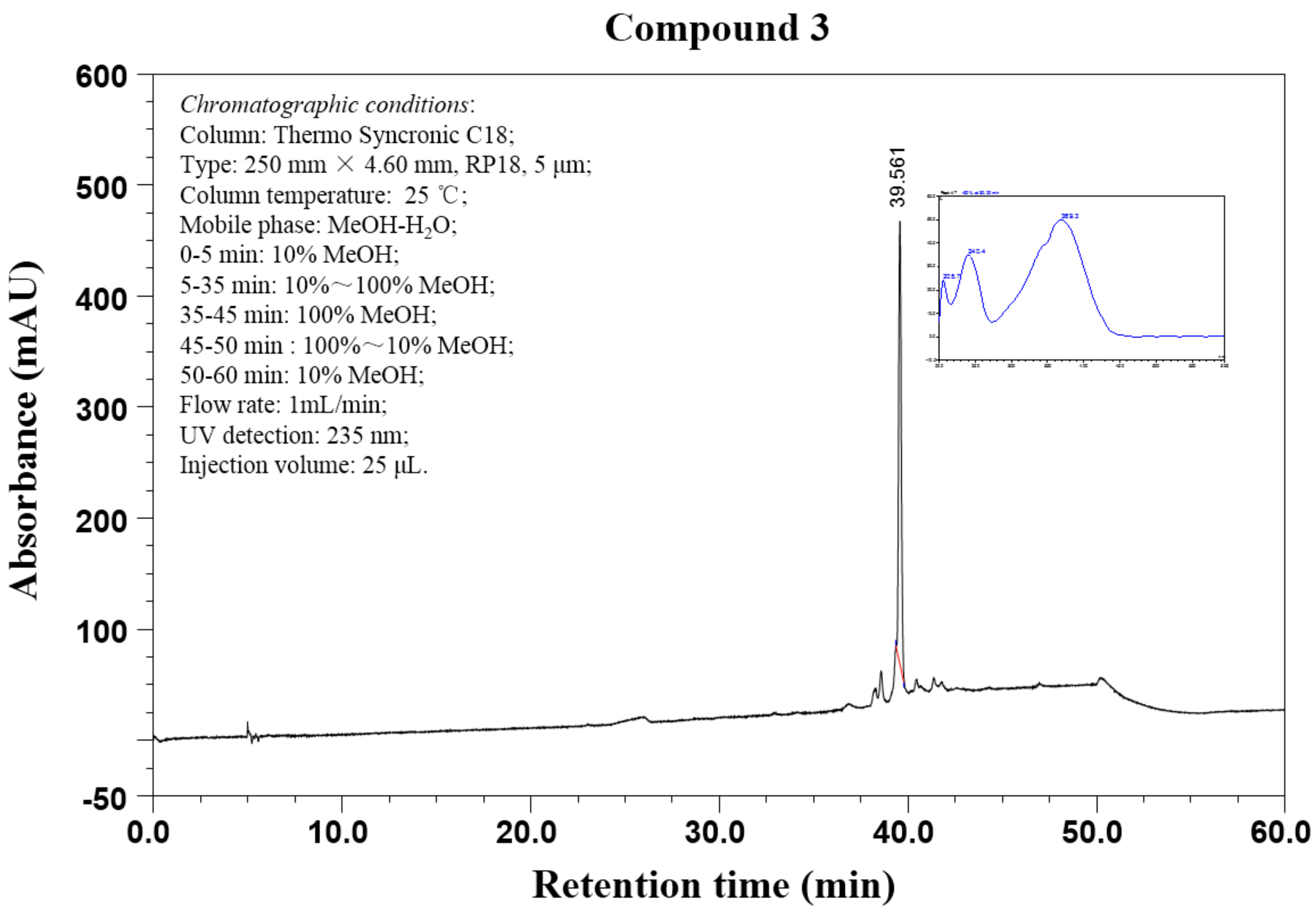

Figure S26. ECD spectrum of compound **3**

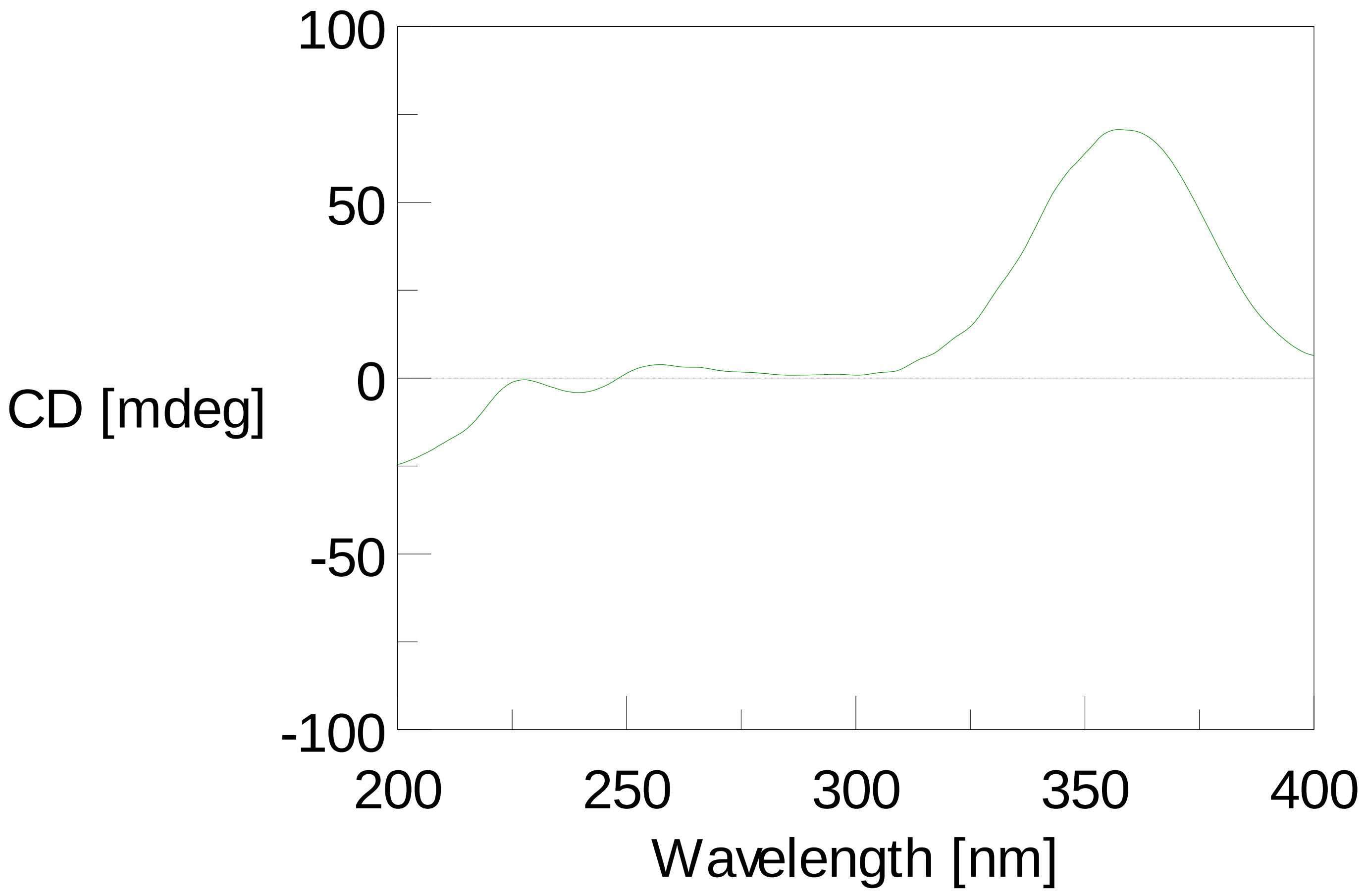

Figure S27. Crystal packing of compound **3** at 293(2) K

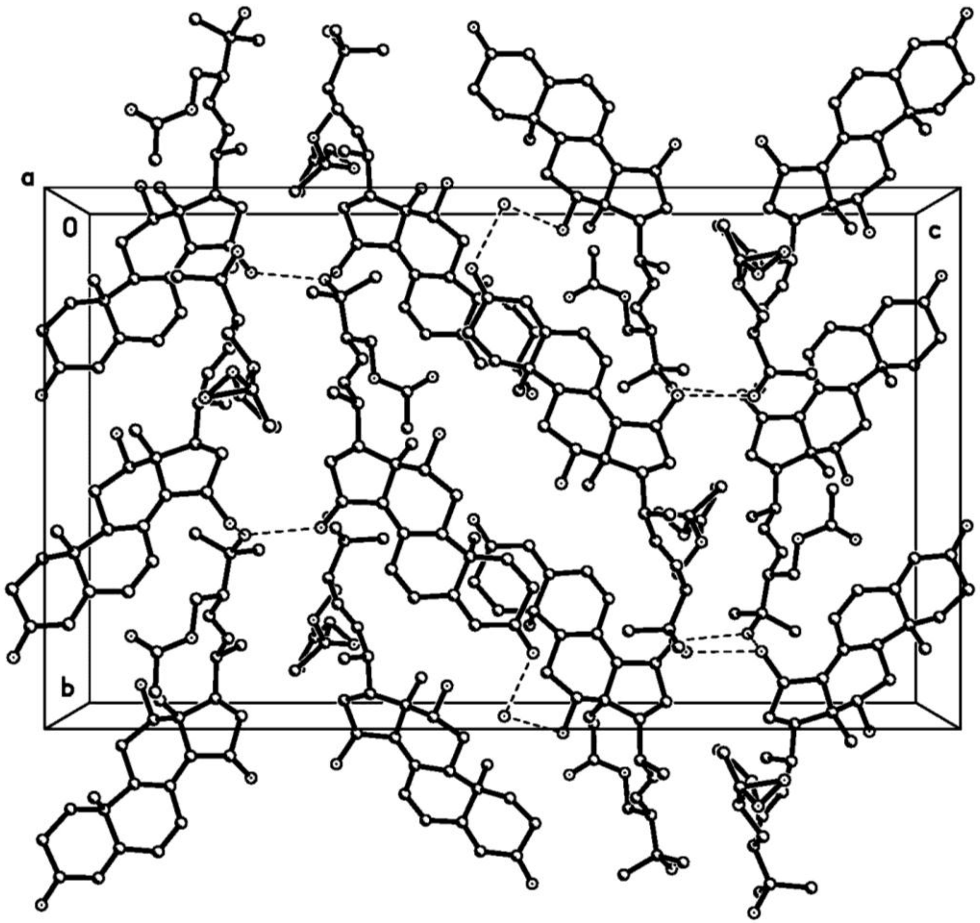

Supplement: Supplementary file 1 [file marinedrugs-16-00114-s001.pdf]
